# Assessment of Intercostal Nerve Block Analgesia for Thoracic Surgery: A Systematic Review and Meta-analysis

**DOI:** 10.1001/jamanetworkopen.2021.33394

**Published:** 2021-11-15

**Authors:** Carlos E. Guerra-Londono, Ann Privorotskiy, Crispiana Cozowicz, Rachel S. Hicklen, Stavros G. Memtsoudis, Edward R. Mariano, Juan P. Cata

**Affiliations:** 1 Department of Anesthesiology and Perioperative Medicine, MD Anderson Cancer Center, University of Texas, Houston; 2 Eastern Virginia Medical School, Norfolk; 3 Department of Anesthesiology, Perioperative Medicine and Intensive Care Medicine, Paracelsus Medical University, Salzburg, Austria; 4 Research Medical Library, MD Anderson Cancer Center, University of Texas, Houston; 5 Department of Anesthesia, Hospital for Special Surgery, New York, New York; 6 Department of Anesthesia, School of Medicine, Stanford University, Stanford, California; 7 Anesthesiology and Surgical Oncology Research Group, Houston, Texas

## Abstract

**Importance:**

The use of intercostal nerve block (ICNB) analgesia with local anesthesia is common in thoracic surgery. However, the benefits and safety of ICNB among adult patients undergoing surgery is unknown.

**Objective:**

To evaluate the analgesic benefits and safety of ICNB among adults undergoing thoracic surgery.

**Data Sources:**

A systematic search was performed in Ovid MEDLINE, Ovid Embase, Scopus, and the Cochrane Library databases using terms for ICNB and thoracic surgery (including *thoracic surgery*, *thoracoscopy*, *thoracotomy*, *nerve block*, *intercostal nerves*). The search and results were not limited by date, with the last search conducted on July 24, 2020.

**Study Selection:**

Selected studies were experimental or observational and included adult patients undergoing cardiothoracic surgery in which ICNB was administered with local anesthesia via single injection, continuous infusion, or a combination of both techniques in at least 1 group of patients. For comparison with ICNB, studies that examined systemic analgesia and different forms of regional analgesia (such as thoracic epidural analgesia [TEA], paravertebral block [PVB], and other techniques) were included. These criteria were applied independently by 2 authors, and discrepancies were resolved by consensus. A total of 694 records were selected for screening.

**Data Extraction and Synthesis:**

This study followed the Preferred Reporting Items for Systematic Reviews and Meta-analyses (PRISMA) reporting guideline. Data including patient characteristics, type of surgery, intervention analgesia, comparison analgesia, and primary and secondary outcomes were extracted independently by 3 authors. Synthesis was performed using a fixed-effects model.

**Main Outcomes and Measures:**

The coprimary outcomes were postoperative pain intensity (measured as the worst static or dynamic pain using a validated 10-point scale, with 0 indicating no pain and 10 indicating severe pain) and opioid consumption (measured in morphine milligram equivalents [MMEs]) at prespecified intervals (0-6 hours, 7-24 hours, 25-48 hours, 49-72 hours, and >72 hours). Clinically relevant analgesia was defined as a 1-point or greater difference in pain intensity score at any interval. Secondary outcomes included 30-day postoperative complications and pulmonary function.

**Results:**

Of 694 records screened, 608 were excluded based on prespecified exclusion criteria. The remaining 86 full-text articles were assessed for eligibility, and 20 of those articles were excluded. All of the 66 remaining studies (5184 patients; mean [SD] age, 53.9 [10.2] years; approximately 59% men and 41% women) were included in the qualitative analysis, and 59 studies (3325 patients) that provided data for at least 1 outcome were included in the quantitative meta-analysis. Experimental studies had a high risk of bias in multiple domains, including allocation concealment, blinding of participants and personnel, and blinding of outcome assessors. Marked differences (eg, crossover studies, timing of the intervention [intraoperative vs postoperative], blinding, and type of control group) were observed in the design and implementation of studies. The use of ICNB vs systemic analgesia was associated with lower static pain (0-6 hours after surgery: mean score difference, −1.40 points [95% CI, −1.46 to −1.33 points]; 7-24 hours after surgery: mean score difference, −1.27 points [95% CI, −1.40 to −1.13 points]) and lower dynamic pain (0-6 hours after surgery: mean score difference, −1.66 points [95% CI, −1.90 to −1.41 points]; 7-24 hours after surgery: mean score difference, −1.43 points [95% CI, −1.70 to −1.17 points]). Intercostal nerve block analgesia was noninferior to TEA (mean score difference in worst dynamic panic at 7-24 hours after surgery: 0.79 points; 95% CI, 0.28-1.29 points) and marginally inferior to PVB (mean score difference in worst dynamic pain at 7-24 hours after surgery: 1.29 points; 95% CI, 1.16 to 1.41 points). The largest opioid-sparing effect of ICNB vs systemic analgesia occurred at 48 hours after surgery (mean difference, −10.97 MMEs; 95% CI, −12.92 to −9.02 MMEs). The use of ICNB was associated with higher MME values compared with TEA (eg, 48 hours after surgery: mean difference, 48.31 MMEs; 95% CI, 36.11-60.52 MMEs) and PVB (eg, 48 hours after surgery: mean difference, 3.87 MMEs; 95% CI, 2.59-5.15 MMEs).

**Conclusions and Relevance:**

In this study, single-injection ICNB was associated with a reduction in pain during the first 24 hours after thoracic surgery and was clinically noninferior to TEA or PVB. Intercostal nerve block analgesia had opioid-sparing effects; however, TEA and PVB were associated with larger decreases in postoperative MMEs, suggesting that ICNB may be most beneficial for cases in which TEA and PVB are not indicated.

## Key Points

**Question:**

Is the use of intercostal nerve block (ICNB) analgesia safe and beneficial for adults undergoing thoracic surgery?

**Findings:**

In this systematic review and meta-analysis of 66 studies including 5184 adult patients undergoing thoracic surgery, the use of ICNB was associated with a clinically and statistically relevant analgesic benefit during the first 24 hours after thoracic surgery, with outcomes that were superior to systemic analgesia and noninferior to other techniques. Although ICNB was associated with a reduction in postoperative opioid consumption, the extent of this reduction was inferior to that of thoracic epidural and paravertebral block analgesia.

**Meaning:**

This study found that ICNB was safe and beneficial for adults undergoing thoracic surgery, providing a reduction in pain during the first 24 hours after thoracic surgery; ICNB may be most beneficial for cases in which thoracic epidural or paravertebral block analgesia are not indicated.

## Teaser

This systematic review and meta-analysis assesses the benefits and safety associated with intercostal nerve block analgesia compared with systemic analgesia and other local analgesia techniques among adults undergoing thoracic surgery.

## Introduction

Acute pain after thoracic surgery is common and severe, and can lead to increased morbidity.^1,2^ In the thorax, nociception travels primarily via the intercostal nerves.^3^ Therefore, blockade of the intercostal nerve is used to provide analgesia after thoracic surgery.^4^ Intercostal nerve blocks (ICNBs) are a common component of multimodal analgesia for thoracic surgery.^5^ Current guidelines suggest that continuous intercostal analgesia is similar to thoracic epidural analgesia (TEA).^6^ Aside from consideration of technical aspects and costs,^7^ the selection of any analgesic approach is typically based on its clinical benefits and disadvantages.^8,9^

In recent years, the use of minimally invasive techniques in thoracic surgery has substantially increased.^10^ This increase coincided with a decrease in the use of TEA and the emergence of fascial plane blocks.^4^ Previous reviews have found that ICNB was superior to systemic analgesia and was associated with reductions in opioid consumption.^11,12^ However, although Joshi et al^12^ recommended TEA and paravertebral block (PVB) as first-line options, Detterbeck et al^11^ questioned the superiority of TEA vs continuous extrapleural techniques. Considering the findings of these previous studies,^4,10,11,12^ we conducted a systematic review and meta-analysis to synthesize the evidence on the benefits and safety of ICNB among adult patients undergoing thoracic surgery.

We hypothesized that the use of ICNB would be associated with superior analgesia and reductions in opioid consumption compared with systemic analgesia alone, while being inferior to PVB and TEA. We also expected ICNB to be superior to systemic analgesia but inferior to PVB or TEA with regard to postoperative complications.

## Methods

This systematic review and meta-analysis followed the Preferred Reporting Items for Systematic Reviews and Meta-analyses (PRISMA) reporting guideline.^13^ The protocol was registered in the PROSPERO database (registration number: CRD42021224783).

Selected sources included observational and experimental studies of adults 18 years and older undergoing any cardiothoracic surgery in which ICNB with local anesthesia was administered via single injection, continuous infusion, or a combination of both techniques in at least 1 group of patients. The use of ICNB was separately compared with both systemic analgesia and different forms of regional analgesia, including TEA and PVB. The coprimary outcomes were acute postoperative pain intensity (dynamic and static) before hospital discharge and opioid consumption. The secondary outcomes were pulmonary function and 30-day postoperative complications.

A systematic literature search was constructed by a medical librarian (R.S.H.). Ovid MEDLINE, Ovid Embase, Scopus, and the Cochrane Library databases were queried using the following natural language and controlled vocabulary terms for ICNB and thoracic surgery: *thoracic surgery*, *thoracic surgical procedures* (including *cardiac surgical procedures*, *mediastinoscopy*, *pulmonary surgical procedures*, *sternotomy*, *thoracoplasty*, *thoracoscopy*, *thoracostomy*, *thoracotomy*, *thymectomy*, *tracheostomy*, *tracheotomy*, *cardiac*, *heart*, *pulmonary*, and *lung*), and ICNB-related terms (including *nerve block*, *intercostal nerves*, *ICNB*, *nerve*, and *block*). Records included were limited to human studies published in the English language. A sample electronic search is available on the PROSPERO website.^14^ Case reports, conference abstracts, editorial letters, and pediatric-only studies were excluded. Identification of other unpublished studies was not attempted. The search and results were not limited by date. After deduplication, 694 unique records were identified. The last date of search was July 24, 2020.

Only articles with available full text were included. Records were screened independently by 2 authors (C.E.G.-L. and J.P.C.) based on title and abstract. Discrepancies were resolved by consensus. The 2 authors then obtained full text of the remaining articles to assess eligibility.

### Data Collection Process

Three authors (C.E.G.-L., A.P., and J.P.C.) extracted the data independently using Excel spreadsheets (Microsoft Corp) that were subsequently merged after the data were collected. Attempts were made to contact authors for missing data. The following data were extracted: demographic characteristics of participants (age, sex, and body mass index), type of surgery (sternotomy, thoracoscopy, thoracotomy, and not specified), type of intervention (single-injection, continuous administration, dose administered, and type of local anesthetic used), and comparator groups (eg, placebo, systemic analgesia, TEA, PVB, and erector spinae plane block). Pain was extracted as the worst static or dynamic pain on a validated 10-point scale (verbal, numerical, or visual, with 0 indicating no pain and 10 indicating severe pain) within the following postoperative periods: 0 to 6 hours, 7 to 24 hours, 25 to 48 hours, 49 to 72 hours, and more than 72 hours. Clinically relevant analgesia was defined as a 1-point or greater difference in pain intensity score at any interval.^15^ Opioid consumption was extracted for the same intervals and converted to intravenous morphine milligram equivalents (MMEs).

Nominal data were summarized using proportions, whereas continuous data were reported as means with SDs. Data provided as medians and ranges were converted to means and SDs according to the methods described in Hozo et al.^16^ Data from figures were digitized using WebPlotDigitizer software, version 4.4 (Ankit Rohatgi).^17^ Risk of bias in individual studies was assessed according to the criteria described by the Grading of Recommendations, Assessment, Development and Evaluation (GRADE) Working Group^18^ and was considered for each outcome.

### Statistical Analysis

To provide estimates of intervention outcomes, a quantitative fixed-effects meta-analysis was performed using the meta package in R software, version 3.6.3 (R Foundation for Statistical Computing), when data from at least 3 studies were available for each outcome. Summary estimates, including odds ratios (ORs), mean differences, and 95% CIs, were calculated for each outcome. Heterogeneity was assessed using the *I*^2^ statistic and, when serious heterogeneity was detected, subgroups were investigated to identify potential differences. Qualitative analysis was followed by quantitative analysis using the GRADE criteria to rate the quality of evidence.^19^ Results were interpreted in the context of pooled effect estimates; risk of bias, heterogeneity (measured using the *I*^2^ statistic), imprecision, and indirectness were assessed for each outcome across the respective informing studies. Publication bias was determined by inspection of funnel plots for each outcome. Synthesis results, including information on the quality of evidence, are shown in Table 1.

**Table 1.** Summary of Studies Comparing ICNB Analgesia With Other Regional or Systemic Analgesia Techniques

Source	Type of study	Blinding, yes/no (type of blinding)	Type of surgery	ICNB analgesia (No. of patients)	Comparison analgesia (No. of patients)	Prespecified outcomes	Postoperative follow-up period
de la Rocha and Chambers,^20^ 1984	RCT	No	Thoracotomy	Single injection (5)	Continuous (5); no block (5); TENS (5)	Spirometry	5 d
Orr et al,^21^ 1981	RCT	No	Thoracotomy	Single injection (30)	Cryoanalgesia (15)	Pain intensity (VAS); opioid consumption; hemodynamic parameters	≤8 d
Perttunen et al,^22^ 1995	RCT	No	Thoracotomy	Single injection (15)	Epidural (15); PVB (15)	Pain intensity (VAS and NRS); area of analgesic spread; spirometry; complications; blood-gas analysis; LOS; bupivacaine levels	2 d
Fiorelli et al,^23^ 2020	RCT	Yes (SB)	Thoracotomy	Single injection (30)	ESPB (30)	Pain intensity (NRS); opioid consumption; spirometry; patient satisfaction	2 d
Shafei et al,^24^ 1990	RCT	No	Thoracotomy	Single injection (16)	Cryoanalgesia (31); interpleural (16)	Pain intensity (VAS); opioid consumption; complications	7 d
Bergh et al,^25^ 1966	Prospective	No	Thoracotomy	Single injection (30)	No block (6)	Pain sensitivity; opioid consumption; spirometry; blood-gas analysis^a^	7 d
Bolotin et al,^26^ 2000	RCT	No	Thoracoscopy	Single injection (16)	No block (16)	Pain intensity; hemodynamic parameters; opioid consumption^b^	1.5 h
Dryden et al,^27^ 1993	Randomized crossover	Yes (DB)	Thoracotomy	Continuous (10)	No block (10)	Pain intensity (VAS); opioid consumption; complications	2 d
Chan et al,^28^ 1991	RCT	Yes (DB)	Thoracotomy	Continuous (10)	No block (10)	Pain intensity (VAS); opioid consumption; spirometry; bupivacaine levels	24 h
Toledo-Pereyra and DeMeester,^29^ 1979	RCT	No	Thoracotomy	Single injection (10)	No block (10)	Opioid consumption; spirometry	10 d
Liu et al,^30^ 1995	RCT	Yes (DB)	Thoracotomy	Single injection (9)	No block (11)	Pain intensity (VAS); opioid consumption; spirometry; complications	3 d
Kavanagh et al,^31^ 1994	RCT	Yes (DB)	Not specified	Single injection (15)	No block (15)	Pain intensity (VAS); pain sensitivity; opioid consumption; spirometry; blood-gas analysis^a^	3 d
Zhan et al,^32^ 2017	RCT	No	Thoracotomy	Single injection (15)	No block (15)	Pain intensity (VAS); stress biomarkers; complications; LOS	24 h
Joucken et al,^33^ 1987	RCT	Yes (DB)	Thoracotomy	Single injection (15)	Cryoanalgesia (15); no block (15)	Opioid consumption; blood-gas analysis	1.5 d
Faust and Nauss,^34^ 1976	RCT	No	Thoracotomy	Single injection (17)	No block (17)	Blood-gas analysis; spirometry	45 min
Dowling et al,^35^ 2003	RCT	Yes (DB)	Sternotomy	Continuous (16)	No block (19)	Pain intensity (VAS); opioid consumption; spirometry; LOS; complications	2 d
Delilkan et al,^36^ 1973	Prospective	Yes (DB)	Thoracotomy	Single injection (20)	No block (20)	Opioid consumption; spirometry; physiologic and blood-gas analysis; clinical condition	24 h
Baxter et al,^37^ 1987	RCT	Yes (SB)	Sternotomy	Continuous (20)	No block (20)	Pain intensity; opioid consumption; spirometry; blood-gas analysis; complications^b^	5 d
Carretta et al,^38^ 1996	RCT	No	Thoracotomy	Continuous (10)	No block (20)	Pain intensity (VAS); opioid consumption; spirometry; blood-gas analysis^b^	2 d
Ghafouri et al,^39^ 2008	RCT	No	Thoracotomy	Single injection (25)	No block (25)	Pain intensity; opioid consumption; spirometry^b^	3 d
Kolvenbach et al,^40^ 1989	Prospective	No	Thoracotomy	Continuous (25)	No block (30)	Complications; mortality; bupivacaine levels; patient satisfaction	In-hospital stay
Ahmed et al,^41^ 2017	RCT	Yes (DB)	Thoracoscopy	Single injection (30)	No block (30)	Pain intensity (VAS); opioid consumption	24 h
Barr et al,^42^ 2007	RCT	Yes (DB)	Sternotomy	Single injection (41)	No block (40)	Pain intensity (VAS and NRS); opioid consumption; complications^b^	25 h
Lee et al,^43^ 2019	RCT	Yes (DB)	Sternotomy	Single injection (38)	No block (41)	Pain intensity; opioid consumption; LOS; complications^b^	3 d
Zhu et al,^44^ 2018	RCT	No	Thoracotomy	Single injection (40)	No block (41)	Pain intensity (VAS); opioid consumption; hemodynamic parameters; complications.	48 h
Galway et al,^45^ 1975	RCT	Yes (DB)	Thoracotomy	Single injection (46)	No block (46)	Pain intensity; opioid consumption; hemodynamic parameters; complications^b^	24 h
Wang et al,^46^ 2019	RCT	No	Thoracotomy	Single injection (50)	No block (50)	Pain intensity (VAS); cognitive function; inflammatory biomarkers	24 h
Kaplan et al,^47^ 1975	Prospective	No	Thoracotomy	Single injection (12)	No block (6)	Pain intensity; opioid requirement; spirometry; complications; duration of block; blood-gas analysis; complications^b^	3 d
D’Andrilli et al,^48^ 2006	RCT	No	Thoracotomy	Single injection (60)	No block (60)	Pain intensity (VAS); patient satisfaction; LOS; complications	In-hospital stay, d
Mozell et al,^49^ 1991	RCT	Yes (DB)	Thoracotomy	Continuous (8)	No block (8)	Pain intensity (VAS); opioid consumption; spirometry	5 d
Yang et al,^50^ 2019	Retrospective	NA	Thoracoscopy	Single injection (14)	PVB (14)	Pain (VAS); blood-gas analysis; anesthetic requirement	1 h
Xia et al,^51^ 2020	Retrospective	NA	Thoracoscopy	Single injection (20)	PVB (20)	Pain (VAS); opioid consumption; blood-gas analysis; complications	2 d
Hutchins et al,^52^ 2017	RCT	No	Thoracoscopy	Single injection (25)	PVB (23)	Pain intensity (NRS); opioid consumption; LOS; patient satisfaction; complications	2 d
Chen et al,^53^ 2020	RCT	Yes (DB)	Thoracoscopy	Single injection (24)	ESPB (24); PVB (24)	Pain intensity (VAS); opioid consumption; complications	2 d
Kadomatsu et al,^54^ 2018	RCT	No	Thoracoscopy	Continuous (24)	PVB (26)	Pain intensity (VAS); complications	2 d
Matyal et al,^55^ 2015	Prospective	No	Thoracoscopy	Single injection (20)	PVB (30)	Pain intensity (VAS); opioid consumption; spirometry; LOS	2 mo
Wu et al,^56^ 2018	RCT	Yes (SB)	Thoracoscopy	Single injection (32)	PVB (34)	Pain intensity (VAS); opioid consumption; time to ambulation; complications	2 d
Mogahed and Elkahwagy,^57^ 2020	RCT	No	Thoracoscopy	Single injection (35)	No block (35); PVB (35)	Pain intensity (VAS); spirometry blood-gas analysis; hemodynamic parameters; duration of recovery from anesthesia	2 h
Xiang et al,^58^ 2020	RCT	No	Thoracoscopy	Single injection (40)	No block (40); PVB (40)	Pain intensity (NRS); hemodynamic parameters; blood-gas analysis; procedural cost; patient satisfaction; LOS; complications	24 h
Zheng et al,^59^ 2020	RCT	No	Thoracoscopy	Single injection (50)	PVB (50)	Surgical visualization; duration of the technique; complications	Surgery
Oksuz et al,^60^ 2018	Retrospective	NA	Thoracotomy	Single injection (22)	SAPB (20)	Pain (VAS); opioid consumption; complications	24 h
Kim et al,^61^ 2021	RCT	No	Thoracoscopy	Single injection (25)	SAPB (25)	Pain intensity (NRS); opioid consumption	1-2 d (until chest tube removal)
Hsieh et al,^62^ 2016	Retrospective	NA	Thoracoscopy	Continuous (39)	Single injection (39)	Pain intensity (VAS and NRS); opioid consumption; pulmonary function; LOS	In-hospital stay
Wu et al,^63^ 2016	Retrospective	NA	Thoracoscopy	Continuous (50)	Single injection (50)	Pain (NRS and VAS); opioid consumption; LOS	In-hospital stay
Bachmann-Mennenga et al,^64^ 1993	RCT	No	Thoracotomy	Single injection (10)	Interpleural (10); no block (10); TEA (10)	Pain intensity; hemodynamic parameters; stress biomarkers; blood-gas analysis; bupivacaine levels^b^	6 h
Concha et al,^65^ 2004	RCT	No	Thoracotomy	Single injection (16)	TEA (15)	Pain intensity (VAS); opioid consumption, spirometry	2 d
Kaiser et al,^66^ 1998	RCT	No	Thoracotomy	Continuous (15)	TEA (15)	Pain intensity; opioid consumption; spirometry; mortality; complications; bupivacaine levels^b^	5 d
Wurnig et al,^67^ 2002	RCT	No	Thoracotomy	Single injection (15)	TEA (15)	Pain intensity (VAS); opioid consumption; procedural cost; complications	6 d
Scheinin et al,^68^ 1987	RCT	No	Thoracotomy	Single injection (10)	Preincisional single injection (11); TEA (18)	Pain intensity (VAS); opioid consumption; blood-gas analysis; spirometry; stress biomarkers; bupivacaine levels	24 h
Pompeo et al,^69^ 2013	Prospective	No	Thoracoscopy	Single injection (10)	TEA (20)	Pain intensity (VAS); technique feasibility; blood-gas analysis; hemodynamic parameters; procedural costs	In-hospital stay
Takamori et al,^70^ 2002	RCT	No	Thoracotomy	Single injection + TEA (20)	TEA (20)	Pain intensity (VAS); analgesic consumption; food intake; stress biomarkers^b^	5 d
Ueda et al,^71^ 2020	RCT	No	Thoracoscopy	Single injection (21)	TEA (22)	Pain intensity (VAS); spirometry; 6-min walking distance; duration of technique; opioid consumption; complications	7 d
Mehran et al,^72^ 2017	Retrospective	NA	Thoracoscopy and Thoracotomy	Single injection (247)	TEA (247)	Complications; LOS; mortality	In-hospital stay
Debreceni et al,^73^ 2003	RCT	Yes (DB)	Thoracotomy	Continuous (22)	TEA (25)	Pain intensity (VAS); opioid consumption; blood-gas analysis; hemodynamic parameters; spirometry	20 h
Vilvanathan et al,^74^ 2020	RCT	No	Thoracotomy	Single injection (25)	TEA (25)	Pain intensity (NRS); motor blockade scale; opioid consumption; complications	24 h
Chen et al,^75^ 2018	Retrospective	NA	Thoracoscopy	Single injection (135)	No block (772); TEA (255)	LOS; postoperative cough	12 mo
Ranganathan et al,^76^ 2020	RCT	Yes (DB)	Thoracotomy	Single injection plus TEA (29)	TEA (30)	Pain intensity (NRS); opioid consumption; spirometry	24 h
Sagiroglu et al,^77^ 2013	RCT	Yes (DB)	Thoracotomy	Continuous (30)	TEA (30)	Pain intensity (VAS); opioid consumption; hemodynamic parameters; complications	24 h
Asantila et al,^78^ 1986	RCT	No	Thoracotomy	Single injection (10)	Repeated single injection (10); TEA (31)	Pain intensity (VAS); opioid consumption; blood-gas analysis; spirometry	24 h
Khalil et al,^79^ 2015	Retrospective	NA	Thoracotomy	Single injection (53)	TEA (32)	Pain (VAS); opioid consumption; LOS; complications	3 d
Dauphin et al,^80^ 1997	RCT	No	Thoracotomy	Continuous (31)	TEA (41)	Pain intensity (VAS); opioid consumption; bupivacaine levels	3 d
Meierhenrich et al,^81^ 2011	RCT	No	Thoracotomy	Single injection (42)	TEA (41)	Pain intensity; opioid consumption; spirometry; LOS; complications	In-hospital stay
Luketich et al,^82^ 2005	RCT	No	Thoracotomy	Continuous (47)	TEA (44)	Pain intensity (VAS); opioid consumption; spirometry; technique success rate; LOS^b^	6 d
Rice et al,^83^ 2015	Retrospective	NA	Thoracoscopy and Thoracotomy	Single injection (54)	TEA (54)	Pain (NRS); opioid consumption; complications	In-hospital stay
Hung et al,^84^ 2015	Retrospective	NA	Thoracoscopy	Single injection (108)	TEA (130)	Pain (VAS); mortality; conversion to intubation; complications; LOS; duration of anesthesia/surgery; blood-gas analysis; intraoperative hemodynamic parameters	In-hospital stay
Ambrogi et al,^85^ 2014	Retrospective	NA	Thoracoscopy	Single injection (20)	TEA (20)	Pain intensity (VAS); conversion to general anesthesia; spirometry; blood-gas analysis; hemodynamic parameters; complications	In-hospital stay

Abbreviations: DB, double-blind; ESPB, erector spinae plane block; ICNB, intercostal nerve block; LOS, length of stay; NA, not applicable; NRS, numeric rating scale; PVB, paravertebral block; RCT, randomized clinical trial; SAPB, serratus anterior plane block; SB, single-blind; TEA, thoracic epidural analgesia; TENS, transcutaneous electrical nerve stimulation; VAS, visual analog scale.

^a^ Tactile pain thresholds.

^b^ Includes the use of a scale other than NRS or VAS.

To synthesize data, the Cochrane criteria^86^ were used for randomized clinical trials, and the GRADE criteria^19^ were used for nonrandomized and observational studies. To evaluate the quality of the body of evidence for each individual outcome according to GRADE criteria, the seriousness of risk was assessed across all informing studies to grade the certainty of evidence for each effect estimate.^18^

## Results

### Study Selection

**Figure 1.**
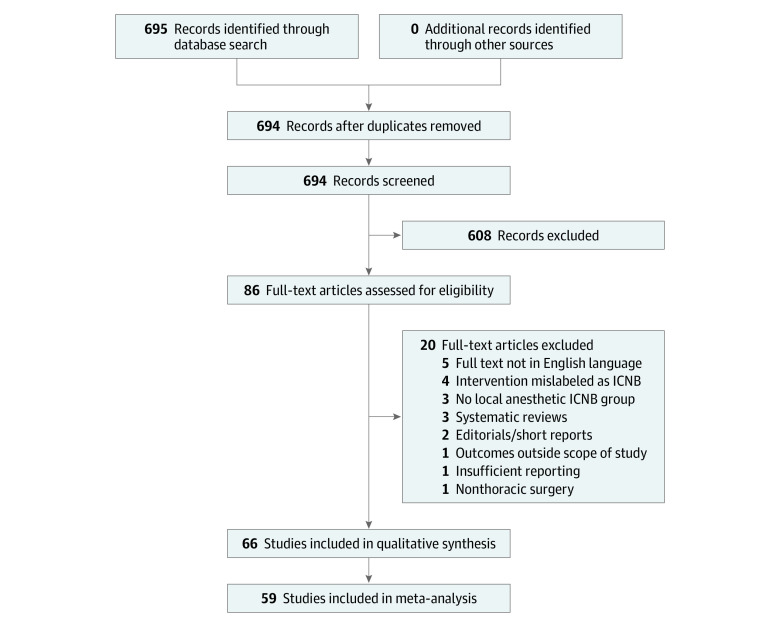
PRISMA Flow Diagram

Of 694 records screened, 608 were excluded based on the prespecified exclusion criteria discussed in Methods (Figure 1). The remaining 86 full-text articles were assessed for eligibility, and 20 of those articles were excluded (5 were not written in the English language, 4 included interventions that were mislabeled as ICNB, 3 did not include a group of patients who received ICNB with local anesthesia, 3 were systematic reviews, 2 were editorials or short reports, 1 examined outcomes outside the scope of the present study, 1 had insufficient reporting, and 1 examined nonthoracic surgery). All of the remaining 66 studies^20,21,22,23,24,25,26,27,28,29,30,31,32,33,34,35,36,37,38,39,40,41,42,43,44,45,46,47,48,49,50,51,52,53,54,55,56,57,58,59,60,61,62,63,64,65,66,67,68,69,70,71,72,73,74,75,76,77,78,79,80,81,82,83,84,85^ (5184 patients; mean [SD] age, 53.9 [10.2] years; approximately 59% men and 41% women) were included in the qualitative analysis; of those, 59 studies^20,22,25,26,28,29,30,31,32,33,34,36,37,38,39,40,41,42,44,45,46,47,48,49,50,51,52,53,54,55,56,57,58,59,65,66,67,68,69,71,72,73,74,77,78,79,80,81,83,84^ (3325 patients) that provided data for at least 1 outcome were included in the quantitative meta-analysis.

### Qualitative Synthesis

Among 66 studies^20,21,22,23,24,25,26,27,28,29,30,31,32,33,34,35,36,37,38,39,40,41,42,43,44,45,46,47,48,49,50,51,52,53,54,55,56,57,58,59,60,61,62,63,64,65,66,67,68,69,70,71,72,73,74,75,76,77,78,79,80,81,82,83,84,85^ included in the qualitative analysis, 55 studies^20,21,22,23,24,25,26,27,28,29,30,31,32,33,34,35,36,37,38,39,40,41,42,43,44,45,46,47,48,49,52,53,54,55,56,57,58,59,61,64,65,66,67,68,69,70,71,73,74,76,77,78,80,81,82^ (3024 patients) were experimental, and 11 studies^50,51,60,62,63,72,75,79,83,84,85^ (2160 patients) were observational. Because analgesic techniques vary based on the type of surgery performed, we divided the populations accordingly. Thirty-nine studies^20,21,22,23,24,25,27,28,29,30,32,33,34,36,38,39,40,44,45,46,47,48,49,60,64,65,66,67,68,70,73,74,76,77,78,79,80,81,82^ (1805 patients) examined thoracotomy, 20 studies^26,41,50,51,52,53,54,55,56,57,58,59,61,62,63,69,71,75,84,85^ (2512 patients) examined thoracoscopy, 4 studies^35,37,42,43^ (235 patients) examined sternotomy, 2 studies^72,83^ (602 patients) examined both thoracotomy and thoracoscopy, and 1 study^31^ (30 patients) did not specify the type of thoracic surgery examined (Table 1).

The intervention used was single-injection ICNB in 51 studies^20,21,22,23,24,25,26,29,30,31,32,33,34,36,39,41,42,43,44,45,46,47,48,50,51,52,53,55,56,57,58,59,60,61,64,65,67,68,69,70,71,72,74,75,76,78,79,81,83,84,85^ (4690 patients) and continuous ICNB in 15 studies^27,28,35,37,38,40,49,54,62,63,66,73,77,80,82^ (494 patients). The comparison groups received TEA, interpleural analgesia, intercostal cryoanalgesia, transcutaneous electrical stimulation, PVB, erector spinae plane block, or serratus anterior plane block. The systemic analgesia group comprised patients in treatment arms who received any form of systemic analgesia (with or without placebo) and did not receive any form of regional analgesia (eg, TEA, PVB, or ICNB).

The risk of bias assessments across studies and for each of the coprimary outcomes are provided in eFigure 1 and eFigure 2 in the Supplement. Most experimental studies had a high risk of bias for allocation concealment,^20,21,22,24,25,26,28,30,32,33,34,35,36,37,38,39,40,42,43,44,45,46,47,48,49,52,54,55,57,58,59,64,65,66,67,68,69,70,71,74,78,82^ blinding of outcome assessors,^29,32,44,46,58,59,63,67,71,74^ blinding of participants and personnel,^29,32,44,46,58,59,63,67,71,74^ and other sources of bias,^29,46,59,67,71^ such as study design (eg, crossover studies and studies that used unvalidated pain measures). In 22 experimental studies^21,22,24,25,29,33,38,40,45,49,52,55,61,67,68,69,70,71,73,74,80,81^ (40.0%), a high risk of incomplete data for all outcomes was detected, primarily because of the lack of an intention-to-treat analysis. Most observational studies had a high risk of bias for inadequate control of confounding^51,60,62,75,79,84,85^ and a low risk of bias for incomplete follow-up^50,51,62,63,72,75,79,83,84^ and flawed measurement of exposure.^50,51,62,63,72,75,79,83,84,85^

### Quantitative Synthesis

**Figure 2.**
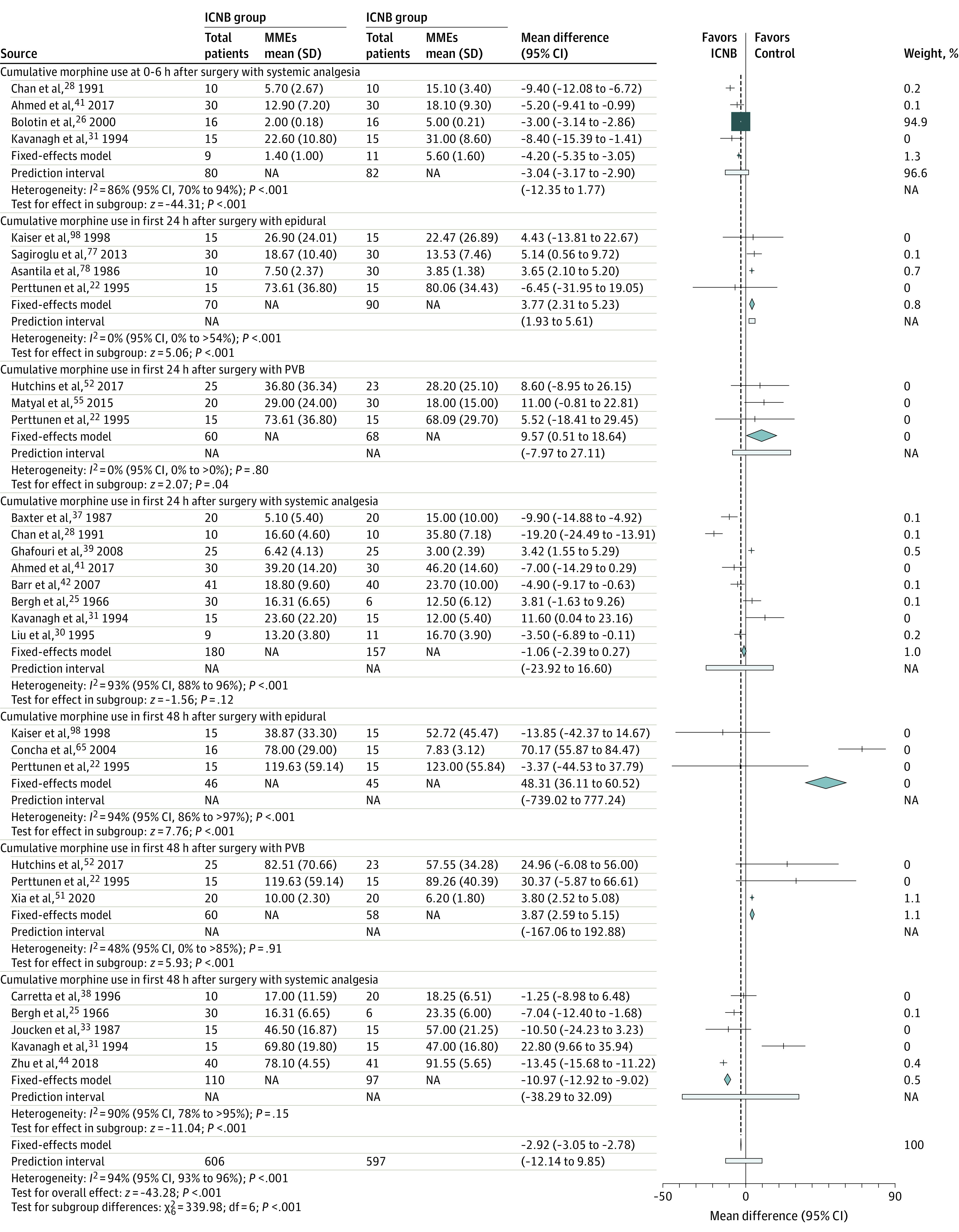
Effect Estimates of Mean Differences in Postoperative MMEs Between Intercostal Nerve Block Analgesia and Other Forms of Analgesia Diamonds represent the results of the fixed-effects model. The size of the squares reflects the weight of each study in the meta-analysis. ICNB indicates intercostal nerve block; MME morphine milligram equivalent; NA, not applicable; and PVB, paravertebral block.

Among 59 studies^20,22,25,26,28,29,30,31,32,33,34,36,37,38,39,40,41,42,44,45,46,47,48,49,50,51,52,53,54,55,56,57,58,59,65,66,67,68,69,71,72,73,74,77,78,79,80,81,83,84^ included in the quantitative meta-analysis, 54 studies^20,22,25,26,28,29,30,31,32,33,34,36,37,38,39,40,41,42,44,45,46,47,48,49,52,53,54,55,56,57,58,59,65,66,67,68,69,71,73,74,77,78,79,80,81^ (2615 patients) were experimental, and 5 studies^50,51,72,83,84^ (710 patients) were observational. Forest plots summarizing the coprimary outcomes are provided in Figure 2 and eFigure 3 in the Supplement. A summary of findings in Table 2 shows the evidence profile and the specific grading of the level of certainty for each outcome. The certainty of evidence for most outcomes was downgraded for reasons including risk of bias, heterogeneity, and imprecision.

**Table 2.** Evidence Profile for the Use of Intercostal Nerve Block Analgesia in Adults Undergoing Thoracic Surgery

Pain outcome^a^	Comparison	Limitations	Risk of bias (domains)^b^	Heterogeneity		Indirectness	Imprecision	Publication bias	Mean difference (95% CI)	No. of participants (No. of studies)	Certainty of evidence (GRADE domains)^c^
				*I*^2^ (95% CI)	*P* value						
Static pain at 0-6 h (11 RCTs^28,30,31,32,41,44,46,48,49,57,58^)	Systemic analgesia	Larger differences in patients having esophagectomy^46^ and pleurectomy^49^	High (1, 2, and 3)	97 (95 to 98)	.02	Not detected	Not detected	Not detected	−1.40 (−1.46 to −1.33)	627 (11 studies)	Moderate (a)
Dynamic pain at 0-6 h (3 RCTs^30,44,58^)	Systemic analgesia	Small samples	High (1, 2, and 3)	95 (89 to 98)	<.001	Not detected	Detected	Detected	−1.66 (−1.90 to −1.41)	181 (3 studies)	Very low (a, b, d, and e)
Static pain at 7-24 h (10 RCTs^28,30,31,38,41,44,46,48,49,58^)	Systemic analgesia	7 of 10 studies examined thoracotomy; high heterogeneity in effect estimates	High (1, 2, and 3)	94 (90 to 96)	<.001	Not detected	Not detected	Not detected	−1.27 (−1.40 to −1.13)	557 (10 studies)	Low (a and b)
Dynamic pain at 7-24 h (4 RCTs^30,31,44,58^)	Systemic analgesia	Both groups received systemic analgesia via PCA and a single-injection neuraxial opioid in 1 study^30^	High (1, 2, and 3)	96 (94 to 98)	.34	Not detected	Detected	Detected	−1.43 (−1.70 to −1.17)	211 (4 studies)	Low (a, b, d, and e)
Static pain at 25-48 h (6 RCTs^30,31,38,44,48,49^)	Systemic analgesia	Larger differences in patients receiving esophagectomy^49^	High (1, 2, and 3)	64 (13 to 85)	<.001	Not detected	Detected	Not serious	−0.37 (−0.60 to −0.14)	297 (6 studies)	Very low (a, b, and d)
Dynamic pain at 25-48 h (3 RCTs^30,31,44^)	Systemic analgesia	Small samples; evidence for thoracotomy	High (1, 2, and 3)	91 (78 to 97)	.02	Not detected	Detected	Not detected	0.51 (0.03 to 0.98)	131 (3 studies)	Low (a, b, and d)
Static pain at 49-72 h (3 RCTs^30,31,49^)	Systemic analgesia	Small sample; most evidence for thoracotomy	High (1)	95 (87 to >98)	<.001	Not detected	Detected	Detected	1.51 (0.94 to 2.08)	66 (3 studies)	Very low (a, b, d, and e)
Static pain at 0-6 h (9 RCTs^22,65,68,69,73,74,77,78,80^)	TEA	8of 9 studies examined thoracotomy	High (1, 2, 3, and 4)	41 (0 to 73)	.09	Not detected	Not detected	Not detected	0.49 (0.18 to 0.79)	389 (9 studies)	Moderate (a)
Dynamic Pain at 0-6 h (4 RCTs^22,65,74,77^)	TEA	Small sample; inconsistent results; most evidence for thoracotomy	High (1, 2, 3, and 4)	61 (0 to 87)	.05	Not detected	Detected	Not detected	0.13 (−0.27 to 0.52)	171 (4 studies)	Low (a and d)
Static pain at 7-24 h (10 RCTs and 1 NRSI^22,65,68,69,71,73,74,77,78,80,84^)	TEA	Observational evidence included	High (1, 2, 3, 4, 6, and 7)	35 (0 to 68)	.34	Not detected	Not detected	Not serious	0.41 (0.21 to 0.61)	672 (11 studies)	Moderate (a)
Dynamic pain at 7-24 h (4 RCTs^22,65,74,77^)	TEA	All studies examined thoracotomy	High (1, 2, 3, and 4)	34 (0 to 77)	<.001	Not detected	Detected	Not detected	0.79 (0.28 to 1.29)	171 (4 studies)	Low (a and d)
Static pain at 25-48 h (4 RCTs and 1 NRSI^22,65,71,80,84^)	TEA	Observational evidence included	High (1, 2, 3, 4, 6, and 7)	67 (13 to 87)	.21	Not detected	Not detected	Detected	0.16 (−0.04 to 0.37)	414 (5 studies)	Low (a and e)
Static pain at 0-6 h (6 RCTs and 1 NRSI^22,50,53,54,56,57,58^)	PVB	1 of 7 studies examined thoracotomy; all others examined thoracoscopy	High (1, 2, and 3)	97 (95 to 98)	.02	Not detected	Not detected	Not detected	0.22 (0.15 to 0.28)	372 (7 studies)	Moderate (a)
Dynamic pain at 0-6 h (3 RCTs^22,53,58^)	PVB	Few studies; different results for thoracotomy vs thoracoscopy	High (1, 2, and 3)	6 (0 to 90)	.09	Not detected	Detected	Detected	0.89 (0.70 to 1.08)	158 (3 studies)	Very low (a, d, and e)
Static pain at 7-24 h (6 RCTs^22,52,54,56,58,75^)	PVB	1 of 6 studies examined thoracotomy	High (1, 2, and 3)	86 (70 to 93)	<.001	Not detected	Not serious	Not serious	0.83 (0.71 to 0.94)	322 (6 studies)	Low (a and b)
Dynamic pain at 7-24 h (3 RCTs^22,52,54,56,58,75^)	PVB	Few studies; different results for thoracotomy vs thoracoscopy	High (1, 2, and 3)	91 (76 to 97)	.12	Not detected	Detected	Detected	1.29 (1.16 to 1.41)	158 (3 studies)	Very low (a, b, d, and e)
Static pain at 25-48 h (5 RCTs^22,52,53,54,56^)	PVB	Inconsistent findings, with 1 study using continuous PVB vs single-injection ICNB^52^	High (1, 2, and 3)	80 (52 to 91)	.12	Not detected	Detected	Not detected	0.07 (−0.04 to 0.19)	242 (5 studies)	Very low (a, b, and d)
Cumulative opioid consumption (MMEs) at 6 h (5 RCTs^26,28,30,31,41^)	Systemic analgesia	Small samples	Unclear (1)	86 (70 to 94)	<.001	Not detected	Detected	High concern	−3.04 (−3.17 to −2.90)	162 (5 studies)	Very low (a, d, and e)
Cumulative opioid consumption (MMEs) in first 24 h (8 RCTs^25,28,30,31,37,39,41,42^)	Systemic analgesia	2 of 8 studies examined sternotomy	High (1, 2, and 3)	93 (88 to 96)	.80	Not detected	Not detected	Not detected	−1.06 (−2.39 to 0.27)	337 (8 studies)	Low (a and b)
Cumulative opioid consumption (MMEs) in first 48 h (5 RCTs^25,31,33,38,44^)	Systemic analgesia	1 study with additional multimodal regimen in ICNB group^31^	High (1, 2, 3, and 4)	90 (78 to >95)	.91	Indirectness for thoracoscopy	Detected	Detected	−10.97 (−12.92 to −9.02)	207 (5 studies)	Very low (a, b, d, and e)
Cumulative opioid consumption (MMEs) in first 24 h (4 RCTs^22,66,77,78^)	TEA	Small samples	High (1, 2, and 3)	0 (0 to >54)	<.001	Indirectness for thoracoscopy	Detected	Not serious	3.77 (2.31 to 5.23)	160 (4 studies)	Low (a and d)
Cumulative opioid consumption (MMEs) at 48 h (3 RCTs^22,65,66^)	TEA	1 study with TEA group without supplemental systemic opioids	High (1, 2, and 3)	94 (86 to >97)	<.001	Indirectness for thoracoscopy	Detected	Detected	48.31 (36.11 to 60.52)	91 (3 studies)	Very low (a, b, d, and e)
Cumulative opioid consumption (MMEs) in first 24 h (3 RCTs^22,52,55^)	PVB	Small samples	High (1, 2, 3, and 4)	0 (0 to >0)	<.001	Not detected	Detected	Not serious	9.57 (0.51 to 18.64)	128 (3 studies)	Low (a and d)
Cumulative opioid consumption (MMEs) in first 48 h (2 RCTs and 1 NRSI^22,51,52^)	PVB	Small samples	High (1, 2, 3, 4, 6, and 7)	48 (0 to >85)	.15	Not detected	Detected	Detected	3.87 (2.59 to 5.15)	118 (3 studies)	Very low (a, d, and e)

Abbreviations: GRADE, Grading of Recommendations, Assessment, Development and Evaluation; ICNB, intercostal nerve block; MME, morphine milligram equivalent; NRSI, nonrandomized study of therapeutic intervention; PCA, patient-controlled analgesia; PVB, paravertebral block; RCT, randomized clinical trial; TEA, thoracic epidural analgesia; VAS, visual analog scale.

^a^ Pain intensity was assessed using VAS score (range, 0-10 points, with 0 indicating no pain and 10 indicting severe pain).

^b^ Risk of bias domains in RCTs: 1 indicates allocation concealment; 2, blinding of outcome assessors for all outcomes; 3, blinding of participants and personnel for all outcomes; 4, incomplete outcome data for all outcomes; and 5, selective reporting, sequence generation. Risk of bias domains in NRSIs: 6 indicates failure to adequately control for confounding; 7, failure to develop and apply appropriate eligibility criteria; 8, flawed measurement of exposure or outcome; and 9, incomplete follow-up.

^c^ GRADE domains for downgrading the evidence: a indicates risk of bias; b, heterogeneity; c, indirectness; d, imprecision; and e, publication bias.

### Static and Dynamic Pain Intensity

Overall, ICNB was superior to systemic analgesia with regard to static and dynamic pain during the first 24 hours after surgery (eFigure 3A in the Supplement). The largest pain reduction occurred at 0 to 6 hours after surgery for both static pain (mean score difference, −1.40 points; 95% CI, −1.46 to −1.33 points) and dynamic pain (mean score difference, −1.66; 95% CI, −1.90 to −1.41). The benefit of ICNB analgesia decreased progressively over time. Static pain scores were lower in the ICNB group at 25 to 48 hours after surgery (mean score difference, −0.37 points; 95% CI, −0.60 to −0.14 points) and shifted in favor of systemic analgesia after 48 hours (mean score difference, 1.51 points; 95% CI, 0.94-2.08 points). Dynamic pain scores changed in favor of systemic analgesia at 25 to 48 hours after surgery (mean score difference, 0.51 points; 95% CI, 0.03-0.98 points).

In the thoracotomy subgroup, ICNB was superior to systemic analgesia with respect to static pain at 0 to 6 hours (mean score difference, −1.88 points; 95% CI, −2.07 to −1.69 points), 7 to 24 hours (mean score difference, −1.55 points; 95% CI, −1.81 to −1.29 points), and 25 to 48 hours (mean score difference, −0.38 points; 95% CI, −0.62 to −0.15 points) after surgery (eFigure 4 in the Supplement). In the thoracoscopy subgroup, ICNB was superior to systemic analgesia for static pain during the first 6 hours after surgery (mean score difference, −1.33 points; 95% CI, −1.40 to −1.27 points).

Intercostal nerve block analgesia was marginally inferior to TEA with regard to static pain during the first 24 hours after surgery only (0-6 hours: mean score difference, 0.49 points [95% CI, 0.18-0.79 points]; 7-24 hours: mean score difference, 0.41 points [95% CI, 0.21-0.61 points]) (eFigure 3B in the Supplement). For dynamic pain, ICNB was noninferior to TEA at 7 to 24 hours after surgery only (mean score difference, 0.79 points; 95% CI, 0.28-1.29 points). In the thoracotomy subgroup, no substantial differences in pain intensity between ICNB and TEA were observed (eg, dynamic pain at 0-6 hours: mean score difference, 0.13 points [95% CI, −0.27 to 0.52 points]; static pain at 0-6 hours: mean score difference, 0.64 points [95% CI, 0.27-1.02 points]) (eFigure 4 in the Supplement). Data for the thoracoscopy subgroup were available only for static pain at 7 to 24 hours after surgery, with a marginal difference in pain scores favoring TEA (mean score difference, 0.32 points; 95% CI, 0.04-0.60 points) (eFigure 4 in the Supplement).

Intercostal nerve block analgesia was inferior to PVB with regard to dynamic and static pain (eFigure 3B in the Supplement). The largest difference was noted in dynamic pain between 7 and 24 hours after surgery (mean score difference, 1.29 points; 95% CI, 1.16-1.41 points). In the thoracoscopy subgroup, patients who received ICNB had higher static pain scores between 7 and 24 hours after surgery compared with those who received PVB (mean score difference, 0.84 points; 95% CI, 0.72-0.96 points) (eFigure 4 in the Supplement).

### Opioid Consumption

The use of ICNB was associated with an opioid-sparing benefit compared with systemic analgesia. Overall, the reduction in opioid consumption associated with ICNB vs systemic analgesia started within the first 6 hours after surgery (mean difference, −3.04 MMEs; 95% CI, −3.17 to −2.90 MMEs) and peaked at 48 hours after surgery (mean difference, −10.97 MMEs; 95% CI, −12.92 to −9.02 MMEs) (Figure 2). In the thoracotomy subgroup, no difference was noted between ICNB and systemic analgesia during the first 24 hours after surgery (mean difference, 0.26 MMEs; 95% CI, −1.25 to 1.76 MMEs) (eFigure 5 in the Supplement). However, a reduction in opioid consumption was present at 48 hours after surgery (mean difference, −11.73 MMEs; 95% CI, −13.70 to −9.76 MMEs).

Intercostal nerve block analgesia was inferior to TEA with regard to opioid consumption at 24 hours after surgery (mean difference, 3.77 MMEs; 95% CI, 2.31-5.23 MMEs) (Figure 2). This effect was more marked at 48 hours after surgery, during which opioid consumption increased to 48.31 MMEs (95% CI, 36.11-60.52 MMEs). These findings were specific to patients undergoing thoracotomy. The high heterogeneity was explained by the Concha et al^65^ study, in which patients allocated to the TEA group only received opioids epidurally, which may have overestimated the opioid-sparing benefit of TEA.

Intercostal nerve block analgesia was only inferior to PVB at 48 hours after surgery (mean difference, 3.87 MMEs; 95% CI, 2.59-5.15 MMEs) (Figure 2).

### Secondary Outcomes

#### Nausea and Vomiting

Intercostal nerve block analgesia was associated with a reduction in the risk of nausea and vomiting compared with systemic analgesia (OR, 0.44; 95% CI, 0.20-0.94) (eFigure 6 in the Supplement). The opposite result was observed for ICNB vs TEA (OR, 1.60; 95% CI, 0.96-2.66) and PVB (OR, 1.66; 95% CI, 0.96-2.89). In the thoracotomy subgroup, no significant differences were observed between ICNB and TEA (eFigure 7 in the Supplement). In the thoracoscopy subgroup, no significant differences were observed between ICNB and PVB (eFigure 7 in the Supplement).

The risk of cardiovascular complications was similar between ICNB and systemic analgesia (OR, 1.07; 95% CI, 0.44-2.63) (eFigure 6 in the Supplement). However, ICNB was associated with a reduction in the risk of cardiovascular complications compared with TEA analgesia (OR, 0.66; 95% CI, 0.46-0.93). In the thoracotomy subgroup, ICNB was associated with a reduced risk of cardiovascular complications compared with TEA (OR, 0.68; 95% CI, 0.47-0.98) (eFigure 7 in the Supplement). In the thoracoscopy subgroup, ICNB was inferior to PVB (OR, 3.4; 95% CI, 1.27-9.08) (eFigure 7 in the Supplement).

The use of ICNB was associated with a reduction in the risk of arterial hypotension compared with TEA (OR, 0.20; 95% CI, 0.06-0.74) (eFigure 6 in the Supplement). In the thoracotomy subgroup, ICNB was associated with a reduction in the risk of hypotension compared with TEA (OR, 0.21; 95% CI, 0.05-0.87) (eFigure 7 in the Supplement). No data were available for thoracoscopic surgery.

Intercostal nerve block analgesia was associated with a reduction in the risk of pulmonary complications compared with systemic analgesia (OR, 0.45; 95% CI, 0.26-0.79) (eFigure 6 in the Supplement). No difference was noted between ICNB and TEA (OR, 0.86; 95% CI, 0.63-1.18) or PVB (OR, 1.07; 95% CI, 0.25-4.63). In the thoracotomy subgroup, ICNB was superior to systemic analgesia but noninferior to TEA (eFigure 7 in the Supplement). No data were available for thoracoscopic surgery.

With few adverse events reported, no significant difference in risk was noted between ICNB and TEA with regard to 30-day mortality, neurologic complications, catheter or injection site infection, hematoma, pruritus, or urinary retention (eFigure 6 in the Supplement). In addition, no substantial difference in the risk of urinary retention was found between ICNB and PVB. Results remained similar when stratified by type of surgery (eFigure 7 in the Supplement).

#### Pulmonary Function

From baseline, ICNB was associated with higher forced expiratory volume in the first second compared with systemic analgesia at 7 to 24 hours (mean difference, 20.19%; 95% CI, 16.45%-23.93%), 25 to 48 hours (mean difference, 15.75%; 95% CI, 12.35%-19.14%), 49 to 72 hours (mean difference, 19.57%; 95% CI, 16.63%-22.52%), and more than 72 hours (mean difference, 19.75%; 95%CI, 16.32%-23.18%) after surgery (eFigure 8 and eFigure 9 in the Supplement). These results were specific to the thoracotomy subgroup.

Intercostal nerve block analgesia was associated with higher forced vital capacity from baseline compared with systemic analgesia at 7 to 24 hours (mean difference, 10.95%; 95% CI, 8.34%-13.57%) and 25 to 48 hours (mean difference, 8.89%; 95% CI, 6.39%-11.38%) after surgery (eFigure 8 and eFigure 9 in the Supplement). These results were specific to the thoracotomy subgroup. When compared with TEA, no difference was observed in the period of 7 to 24 hours after surgery (mean difference, 3.78%; 95% CI, −2.13% to 9.69%).

#### Length of Stay

Data from 7 studies^22,66,69,71,73,77,84^ (478 participants) did not reveal differences in hospital length of stay with the use of ICNB vs TEA (mean difference, −3.38 hours; 95% CI, −10.75 to 4.00 hours) (eFigure 10 in the Supplement).

In the thoracotomy subgroup, ICNB was associated with an increased length of stay of approximately 14.3 hours (95% CI, 0.15-28.45 hours) vs TEA (eFigure 11 in the Supplement). In the thoracoscopy subgroup, the use of ICNB was associated with a decreased length of stay of approximately −9.97 hours (95% CI, −18.61 to −1.33 hours) vs TEA (eFigure 11 in the Supplement).

A slight increase in the length of stay occurred when ICNB was compared with PVB (mean difference, 5.27 hours; 95% CI, 1.11-9.42 hours) (eFigure 11 in the Supplement). The results remained similar when limited to thoracoscopic procedures.

## Discussion

In this systematic review and meta-analysis, the use of single-injection ICNB among adults undergoing thoracic surgery was associated with a small reduction in pain scores during the first 24 hours after surgery. Intercostal nerve block analgesia was superior to systemic opioid-based analgesia, noninferior to TEA, and marginally inferior to PVB. Because ICNB analgesia was also associated with better pulmonary function and a reduction in the risk of pulmonary complications, these findings were clinically relevant. Although ICNB was associated with reductions in opioid consumption compared with systemic analgesia alone, patients receiving ICNB consumed more opioids than those receiving TEA or PVB. However, caution is warranted when interpreting these findings because the quality of evidence was reduced by the limitations of the included studies.

Unlike previous reviews,^11,12^ our study provided estimates of the strength and duration of analgesic benefits. These estimates may allow clinicians to balance the benefits and harms of regional analgesia. Notably, both pulmonary and cardiovascular complications have been associated with postoperative mortality among patients undergoing thoracic surgery.^10^ Our results bring into question the superiority of TEA with regard to analgesia because the differences were minimal and inconsistent.^87^

The data suggested that the benefit of ICNB analgesia decreases progressively and disappears at 24 to 48 hours after surgery. Reliance on ICNB after this period may result in an abrupt lack of analgesia or rebound pain, represented by higher pain scores at 24 hours after surgery for dynamic pain and 48 hours after surgery for static pain.^88^ This finding is relevant because the severity of acute pain may be the main measure associated with the occurrence of chronic pain.^89^ Notably, the fact that ICNB was noninferior to TEA may underscore the known limitations of TEA, which has reported failure rates of up to 30%.^90^ Nevertheless, the success rate of TEA may be improved by the use of ultrasonography^91^ or the implementation of a preoperative block area.^92^ Comparisons between the use of ICNB with liposomal bupivacaine vs other regional analgesia techniques have only been performed in observational studies.^72,83^ Hussain et al^93^ recently reported that the use of liposomal bupivacaine in peripheral nerve block analgesia was not superior to plain local anesthetic formulations. Therefore, we do not consider the use of liposomal bupivacaine as indicated to provide sustained and beneficial analgesia after thoracic surgery.

Systemic hypotension is a known adverse event associated with TEA.^94^ We found that ICNB was associated with a reduction in the risk of hypotension compared with TEA. Whether TEA-associated hypotension is associated with cardiovascular events is unclear given that neuraxial blockade has not been independently associated with worse cardiovascular outcomes.^95^ Meta-analyses comparing TEA with PVB have also found limited high-quality evidence suggesting that PVB is associated with a lower risk of hypotension than TEA without differences in morbidity or mortality.^9,96^ However, most studies comparing the impact of TEA with that of other techniques have not investigated the incidence of silent events, such as myocardial injury after surgery, which may be associated with postoperative mortality.^97^

### Limitations

This study has several limitations. First, most studies^20,21,22,23,24,25,26,27,28,29,30,31,32,33,34,35,36,37,38,39,40,42,43,44,45,46,47,48,49,51,52,54,55,56,57,58,59,60,61,62,64,65,66,67,68,69,70,71,73,74,75,77,78,79,80,81,82,84,85^ included in the meta-analysis had at least 1 domain at a high risk of bias. In addition to small samples, differences in the protocol designs and types of analgesia produced high heterogeneity and imprecision. Differences in opioid consumption associated with different types of analgesia during certain intervals, such as ICNB vs TEA at 48 hours after surgery, may be overestimated. Second, we were unable to perform subgroup analyses incorporating continuous techniques or extended-release formulations. However, the benefits of extended-release medications have recently come into question,^93^ highlighting the need for pharmacological innovation. Third, the analysis of postoperative complications was limited by the use of observational data and the inadequate outcome definitions provided by the randomized clinical trials included in the meta-analysis. Nevertheless, observational studies can overcome the risk of sampling error associated with small samples.^98^ Fourth, most studies^20,21,23,25,26,28,29,31,33,34,36,38,39,41,46,49,50,55,57,61,62,63,64,65,68,69,70,71,73,76,78,80,82^ did not include complications as a primary or secondary outcome, raising concerns about detection bias.

## Conclusions

This systematic review and meta-analysis found that single-injection ICNB was associated with a reduction in pain during the first 24 hours after thoracic surgery. Within the limitations of the available evidence, ICNB was superior to systemic opioid-based analgesia, noninferior to TEA, and marginally inferior to PVB. Although a small opioid-sparing benefit was found for ICNB alone, TEA and PVB were more favorable when opioid reduction was a consideration. Therefore, ICNB analgesia may be most beneficial for cases in which TEA or PVB are not indicated. Randomized clinical trials with rigorous methodological approaches and a priori outcomes that include safety end points are needed.

## Notes

Supplement. **eFigure 1.** Risk of Bias Assessment Across Experimental and Observational Studies **eFigure 2.** Risk of Bias Assessments Within Each of the Coprimary Outcomes **eFigure 3.** Forest Plots With Effect Estimates of Mean Differences in Acute Postoperative Pain Between ICNB and Other Forms of Analgesia **eFigure 4.** Forest Plots With Effect Estimates of Mean Differences in Acute Postoperative Pain Between ICNB and Other Forms of Analgesia: Subgroup Analysis by Type of Surgery **eFigure 5.** Forest Plots With Effect Estimates of Mean Differences in Postoperative Morphine Milligram Equivalents Between ICNB and Other Forms of Analgesia: Subgroup Analysis by Type of Surgery **eFigure 6.** Forest Plots With Effect Estimates of Odds Ratios for Postoperative Complications Between ICNB and Other Forms of Analgesia **eFigure 7.** Forest Plots With Effect Estimates of Odds Ratios for Postoperative Complications Between ICNB and Other Forms of Analgesia: Subgroup Analysis by Type of Surgery **eFigure 8.** Forest Plots With Effect Estimates of Mean Differences in Postoperative Pulmonary Function Between ICNB and Other Forms of Analgesia **eFigure 9.** Forest Plots With Effect Estimates of Mean Differences in Postoperative Pulmonary Function Between ICNB and Other Forms of Analgesia: Subgroup Analysis by Type of Surgery **eFigure 10.** Forest Plots With Effect Estimates of Mean Differences in Length of Stay Between ICNB and Other Forms of Analgesia **eFigure 11.** Forest Plots With Effect Estimates of Mean Differences in Length of Stay Between ICNB and Other Forms of Analgesia: Subgroup Analysis by Type of Surgery
