# Supplementary material for: Assessment of Intercostal Nerve Block Analgesia for Thoracic Surgery: A Systematic Review and Meta-analysis
Source: JAMA Netw Open. 2021 Nov 15;4(11):e2133394. doi: 10.1001/jamanetworkopen.2021.33394 (PMC8593761; doi:10.1001/jamanetworkopen.2021.33394)
Supplement: Supplement. — eFigure 1. Risk of Bias Assessment Across Experimental and Observational Studies eFigure 2. Risk of Bias Assessments Within Each of the Coprimary Outcomes eFigure 3. Forest Plots With Effect Estimates of Mean Differences in Acute Postoperative Pain Between ICNB and Other Forms of Analgesia eFigure 4. Forest Plots With Effect Estimates of Mean Differences in Acute Postoperative Pain Between ICNB and Other Forms of Analgesia: Subgroup Analysis by Type of Surgery eFigure 5. Forest Plots With Effect Estimates of Mean Differences in Postoperative Morphine Milligram Equivalents Between ICNB and Other Forms of Analgesia: Subgroup Analysis by Type of Surgery eFigure 6. Forest Plots With Effect Estimates of Odds Ratios for Postoperative Complications Between ICNB and Other Forms of Analgesia eFigure 7. Forest Plots With Effect Estimates of Odds Ratios for Postoperative Complications Between ICNB and Other Forms of Analgesia: Subgroup Analysis by Type of Surgery eFigure 8. Forest Plots With Effect Estimates of Mean Differences in Postoperative Pulmonary Function Between ICNB and Other Forms of Analgesia eFigure 9. Forest Plots With Effect Estimates of Mean Differences in Postoperative Pulmonary Function Between ICNB and Other Forms of Analgesia: Subgroup Analysis by Type of Surgery eFigure 10. Forest Plots With Effect Estimates of Mean Differences in Length of Stay Between ICNB and Other Forms of Analgesia eFigure 11. Forest Plots With Effect Estimates of Mean Differences in Length of Stay Between ICNB and Other Forms of Analgesia: Subgroup Analysis by Type of Surgery [file jamanetwopen-e2133394-s001.pdf]

Supplementary Online Content

Guerra-Londono CE, Privorotskiy A, Cozowicz C, et al. Assessment of intercostal nerve block analgesia for thoracic surgery: a systematic review and meta-analysis. *JAMA Netw Open*. 2021;4(11):e2133394. doi:10.1001/jamanetworkopen.2021.33394

eFigure 1. Risk of Bias Assessment Across Experimental and Observational Studies

eFigure 2. Risk of Bias Assessments Within Each of the Coprimary Outcomes

eFigure 3. Forest Plots With Effect Estimates of Mean Differences in Acute Postoperative Pain Between ICNB and Other Forms of Analgesia

eFigure 4. Forest Plots With Effect Estimates of Mean Differences in Acute Postoperative Pain Between ICNB and Other Forms of Analgesia: Subgroup Analysis by Type of Surgery

eFigure 5. Forest Plots With Effect Estimates of Mean Differences in Postoperative Morphine Milligram Equivalents Between ICNB and Other Forms of Analgesia: Subgroup Analysis by Type of Surgery

eFigure 6. Forest Plots With Effect Estimates of Odds Ratios for Postoperative Complications Between ICNB and Other Forms of Analgesia

eFigure 7. Forest Plots With Effect Estimates of Odds Ratios for Postoperative Complications Between ICNB and Other Forms of Analgesia: Subgroup Analysis by Type of Surgery

eFigure 8. Forest Plots With Effect Estimates of Mean Differences in Postoperative Pulmonary Function Between ICNB and Other Forms of Analgesia

eFigure 9. Forest Plots With Effect Estimates of Mean Differences in Postoperative Pulmonary Function Between ICNB and Other Forms of Analgesia: Subgroup Analysis by Type of Surgery

eFigure 10. Forest Plots With Effect Estimates of Mean Differences in Length of Stay Between ICNB and Other Forms of Analgesia

eFigure 11. Forest Plots With Effect Estimates of Mean Differences in Length of Stay Between ICNB and Other Forms of Analgesia: Subgroup Analysis by Type of Surgery

This supplementary material has been provided by the authors to give readers additional information about their work.

© 2021 Guerra-Londono CE et al. *JAMA Network Open*.

eFigure 1. Risk of Bias Assessment Across Experimental (A) and Observational (B) Studies

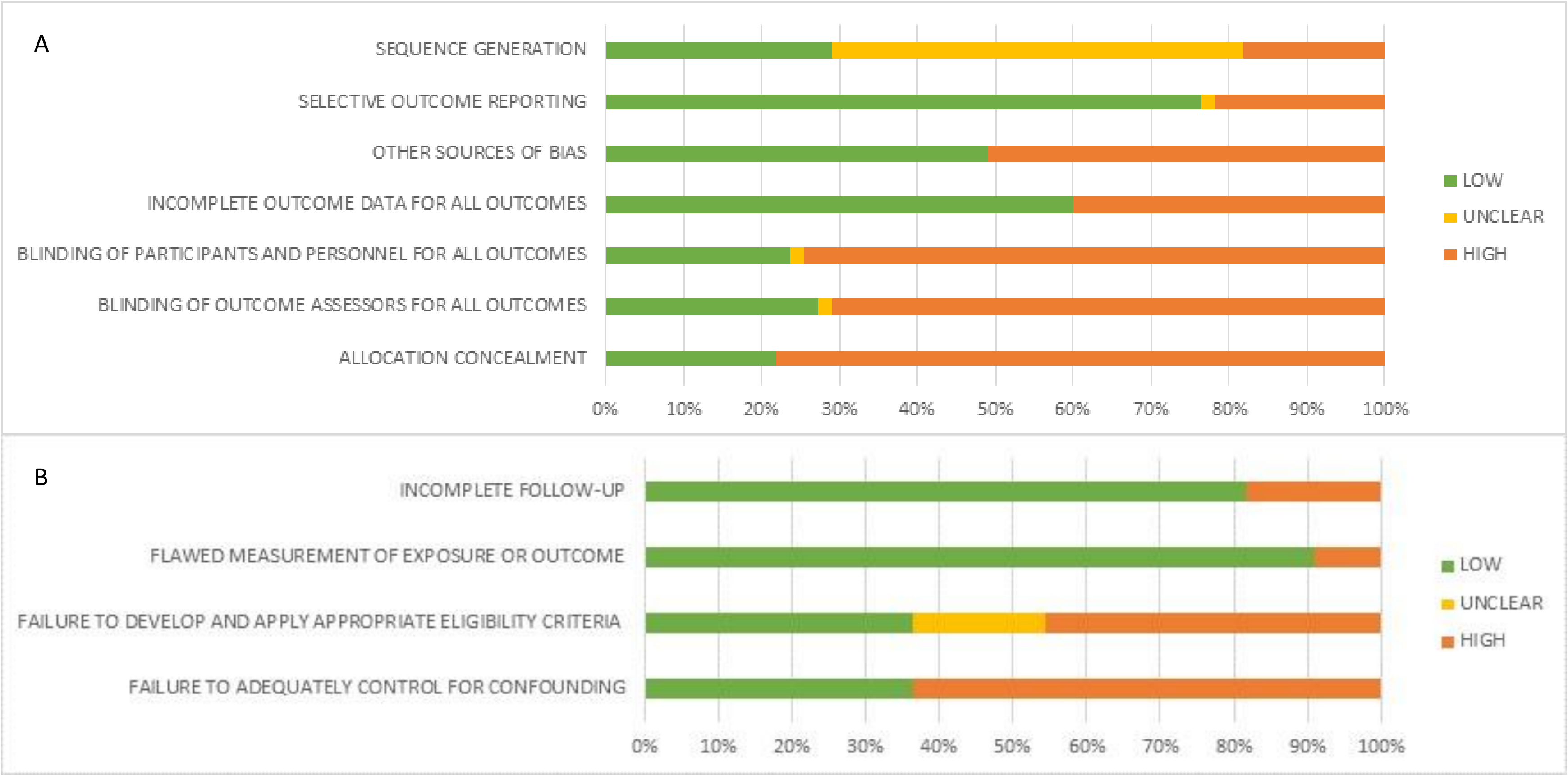

© 2021 Guerra-Londono CE et al. *JAMA Network Open*.

eFigure 2. Risk of Bias Assessments Within Each of the Coprimary Outcomes

A. Risk of Bias Assessment for Pain in Randomized Controlled Trials: ICNB vs Systemic Analgesia

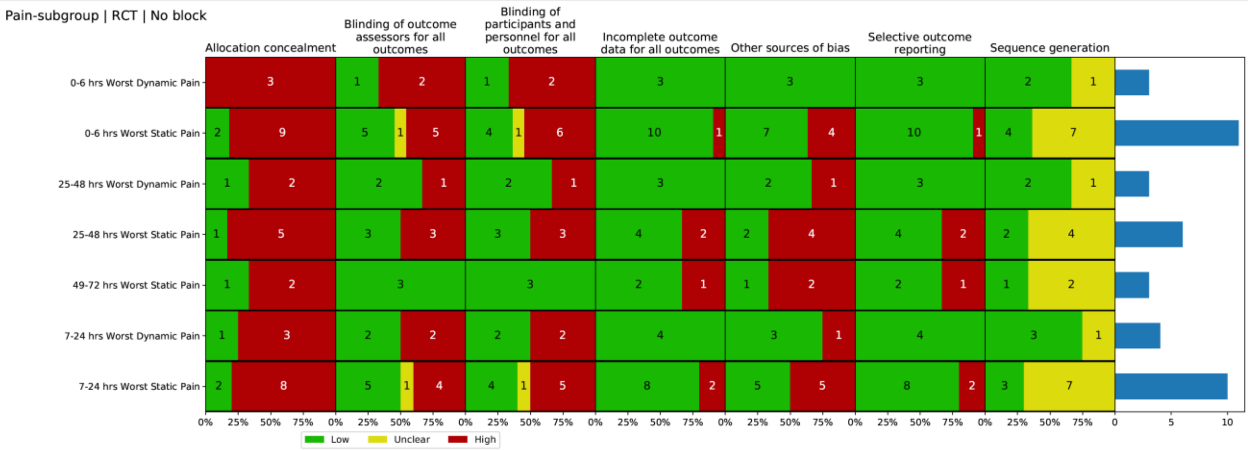

B. Risk of Bias Assessment for Pain in Randomized Controlled Trials: ICNB vs TEA

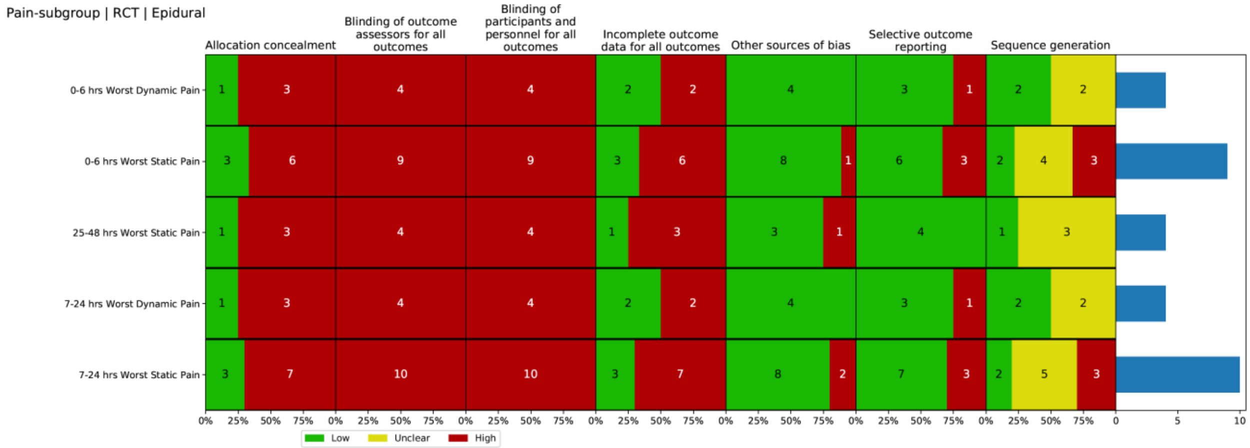

C. Risk of Bias Assessment for Pain in Randomized Controlled Trials: ICNB vs PVB

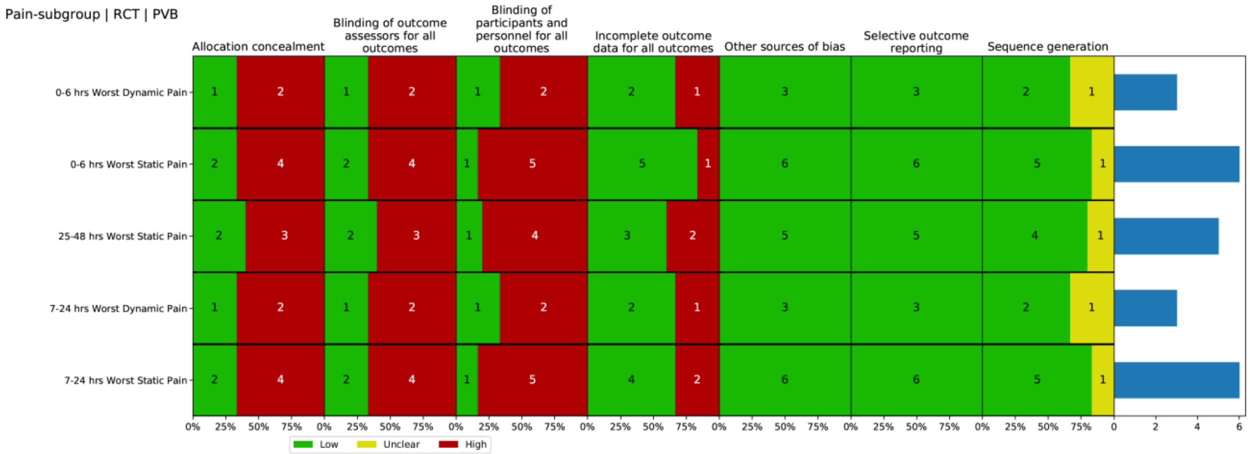

D. Risk of Bias Assessment for Pain in Observational Studies: ICNB vs TEA

© 2021 Guerra-Londono CE et al. *JAMA Network Open*.

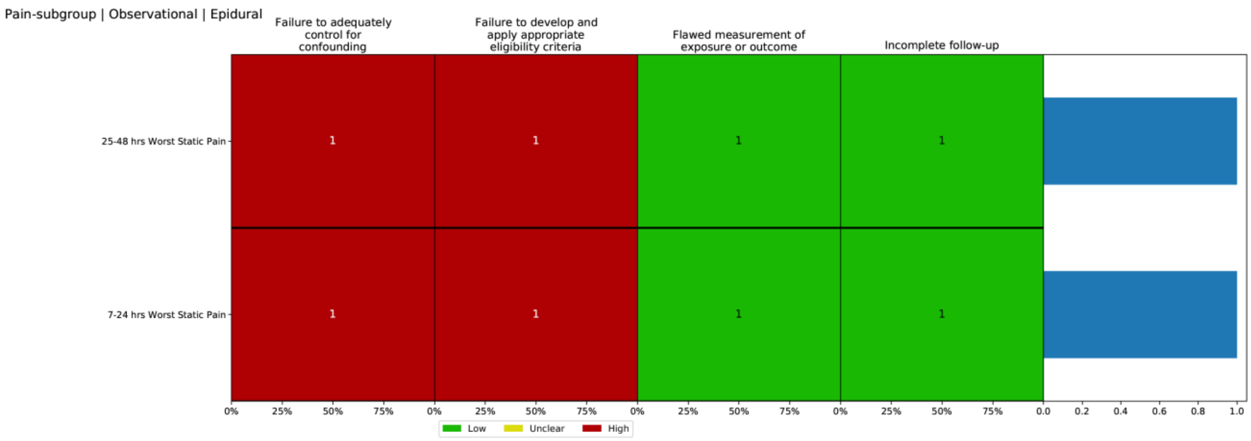

E. Risk of Bias Assessment for Pain in Observational Studies: ICNB vs PVB

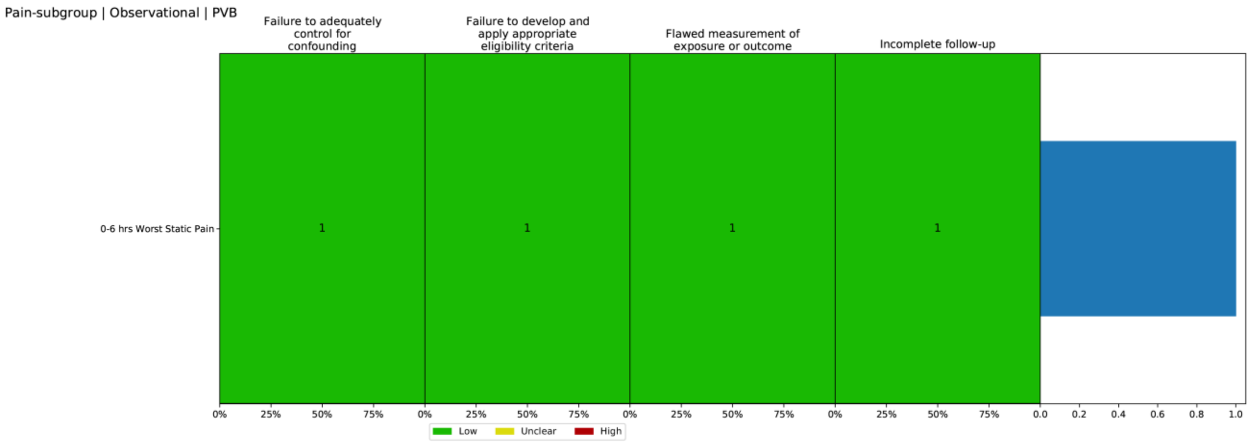

F. Risk of Bias Assessment for Opioid Consumption in Randomized Controlled Trials: ICNB vs Systemic Analgesia

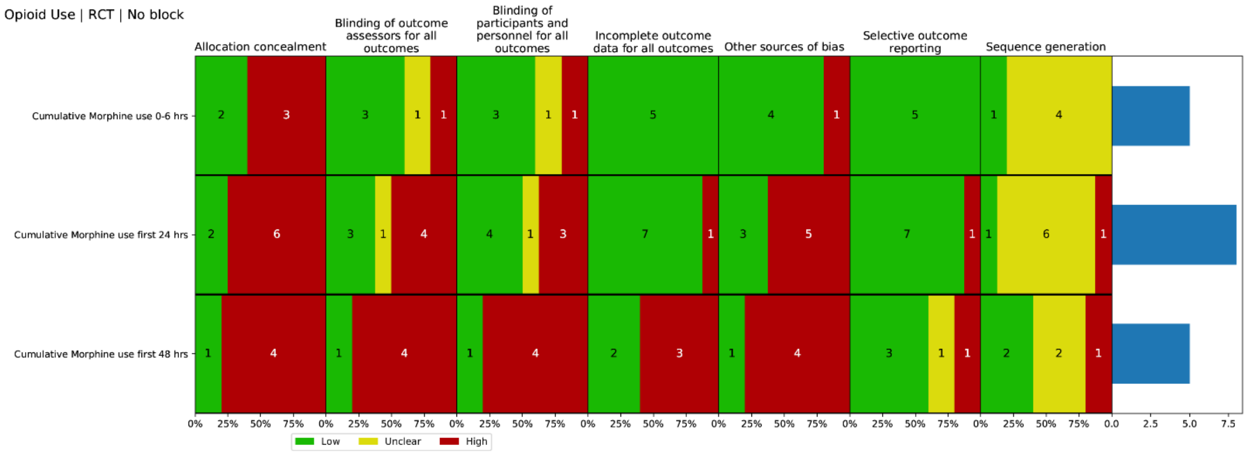

© 2021 Guerra-Londono CE et al. *JAMA Network Open*.

G. Risk of Bias Assessment for Opioid Consumption in Randomized Controlled Trials: ICNB vs TEA

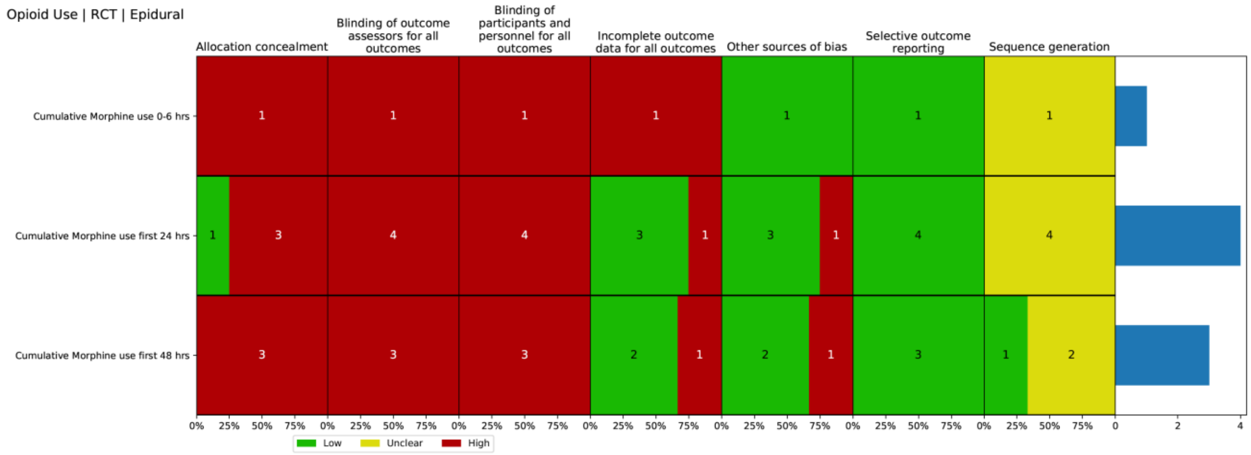

H. Risk of Bias Assessment for Opioid Consumption in Randomized Controlled Trials: ICNB vs PVB

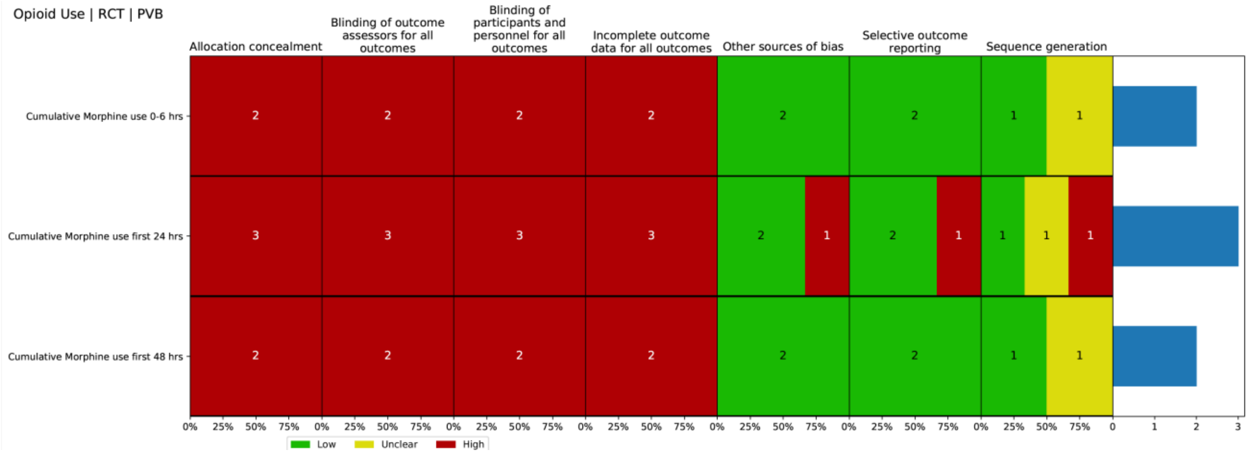

I. Risk of Bias Assessment for Opioid Consumption in Observational Studies: ICNB vs PVB

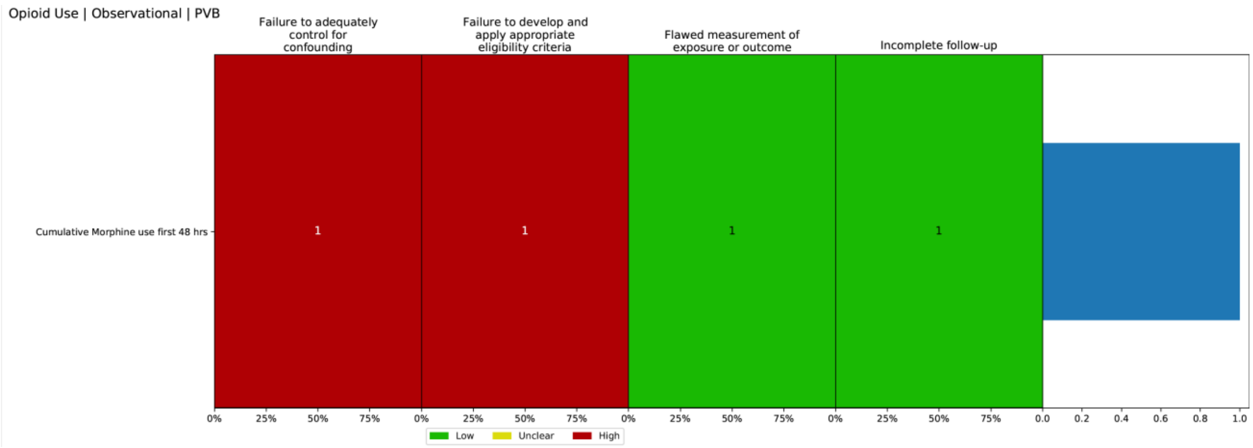

© 2021 Guerra-Londono CE et al. *JAMA Network Open*.

eFigure 3. Forest Plots With Effect Estimates of Mean Differences in Acute Postoperative Pain Between ICNB and Other Forms of Analgesia

A. Forest Plot With Effect Estimates of Mean Differences in Acute Postoperative Pain Between ICNB and Systemic Analgesia (double-click to open PDF)

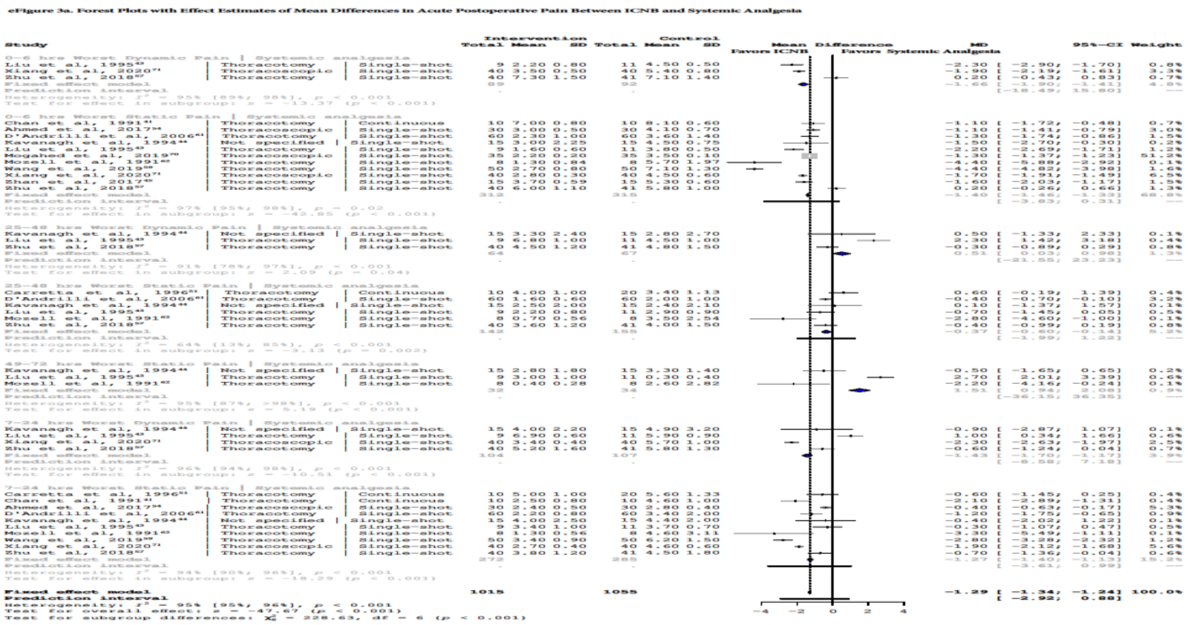

© 2021 Guerra-Londono CE et al. *JAMA Network Open*.

B. Forest Plot With Effect Estimates of Mean Differences in Acute Postoperative Pain Between ICNB and Other Forms of Analgesia (PVB and TEA) (double-click to open PDF)

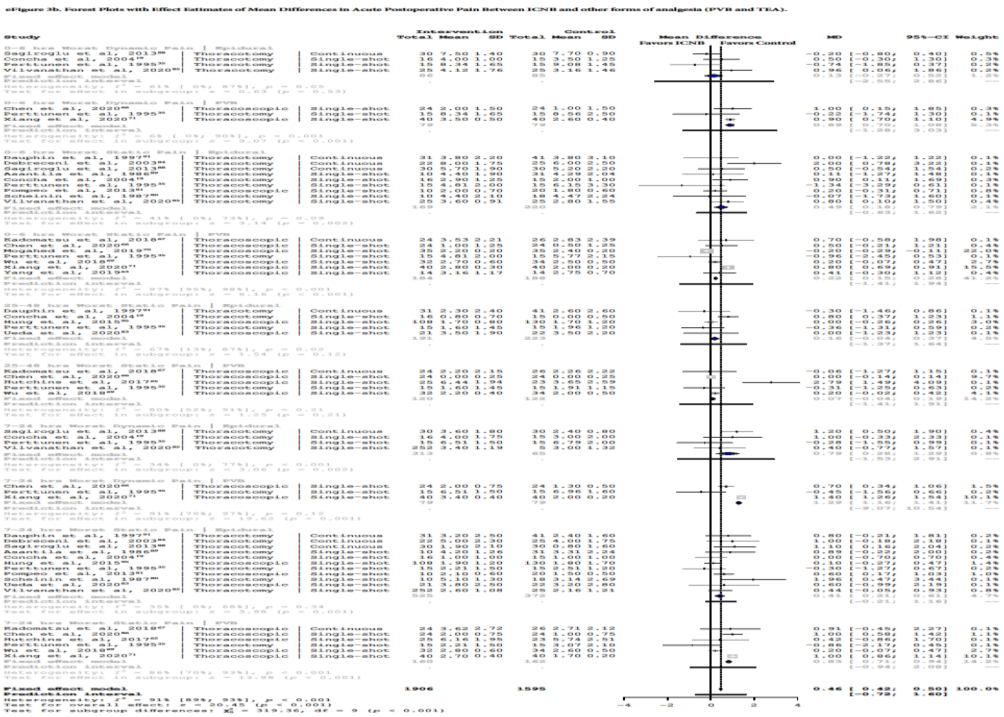

© 2021 Guerra-Londono CE et al. *JAMA Network Open*.

eFigure 4. Forest Plots With Effect Estimates of Mean Differences in Acute Postoperative Pain Between ICNB and Other Forms of Analgesia: Subgroup Analysis by Type of Surgery (double-click to open PDF)

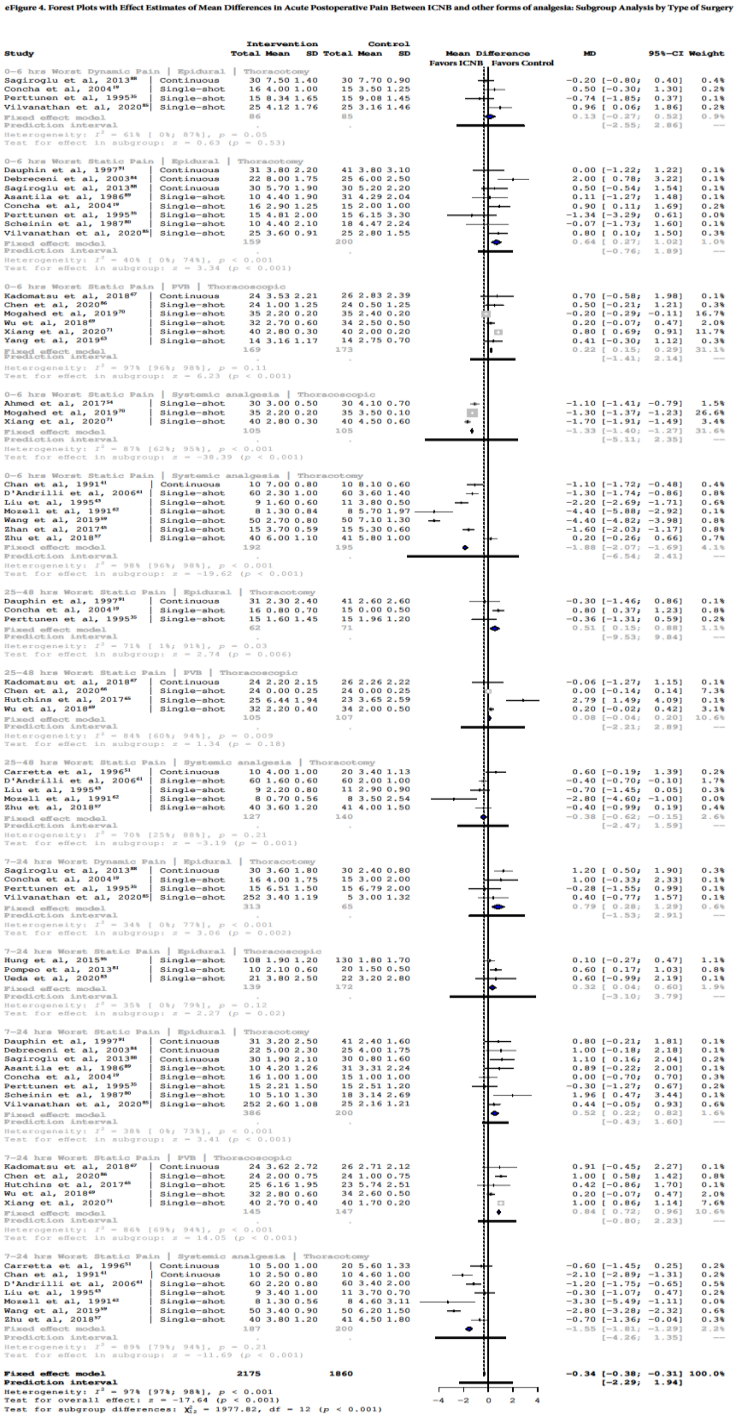

© 2021 Guerra-Londono CE et al. *JAMA Network Open*.

eFigure 5. Forest Plots With Effect Estimates of Mean Differences in Postoperative Morphine Milligram Equivalents Between ICNB and Other Forms of Analgesia: Subgroup Analysis by Type of Surgery (double-click to open PDF)

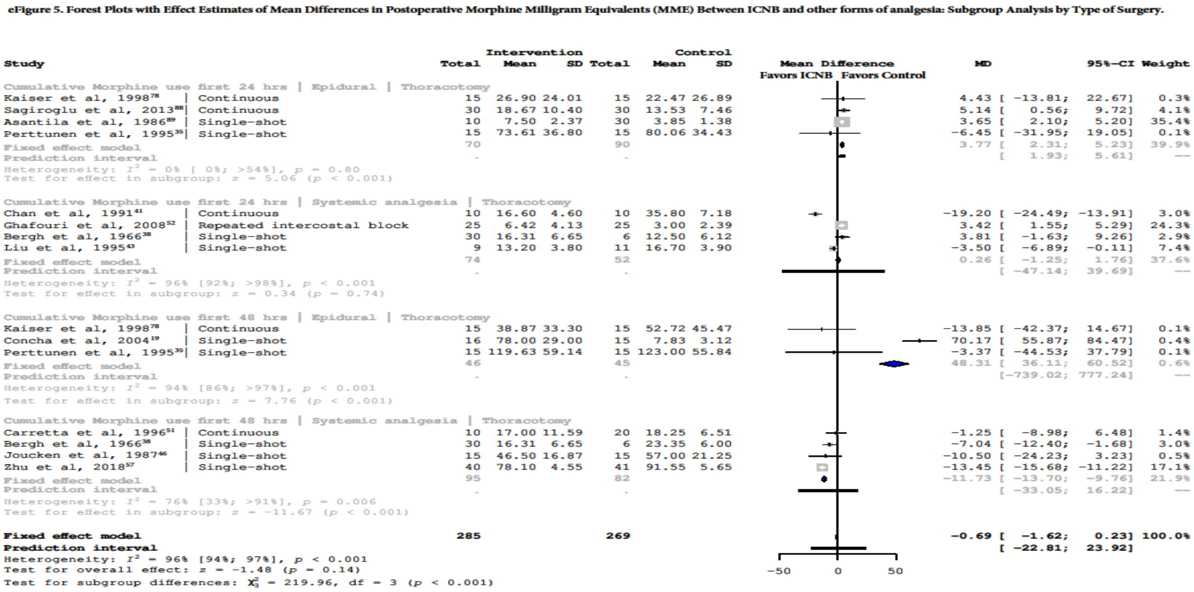

© 2021 Guerra-Londono CE et al. *JAMA Network Open*.

eFigure 6. Forest Plots With Effect Estimates of Odds Ratios for Postoperative Complications Between ICNB and Other Forms of Analgesia (double-click to open PDF)

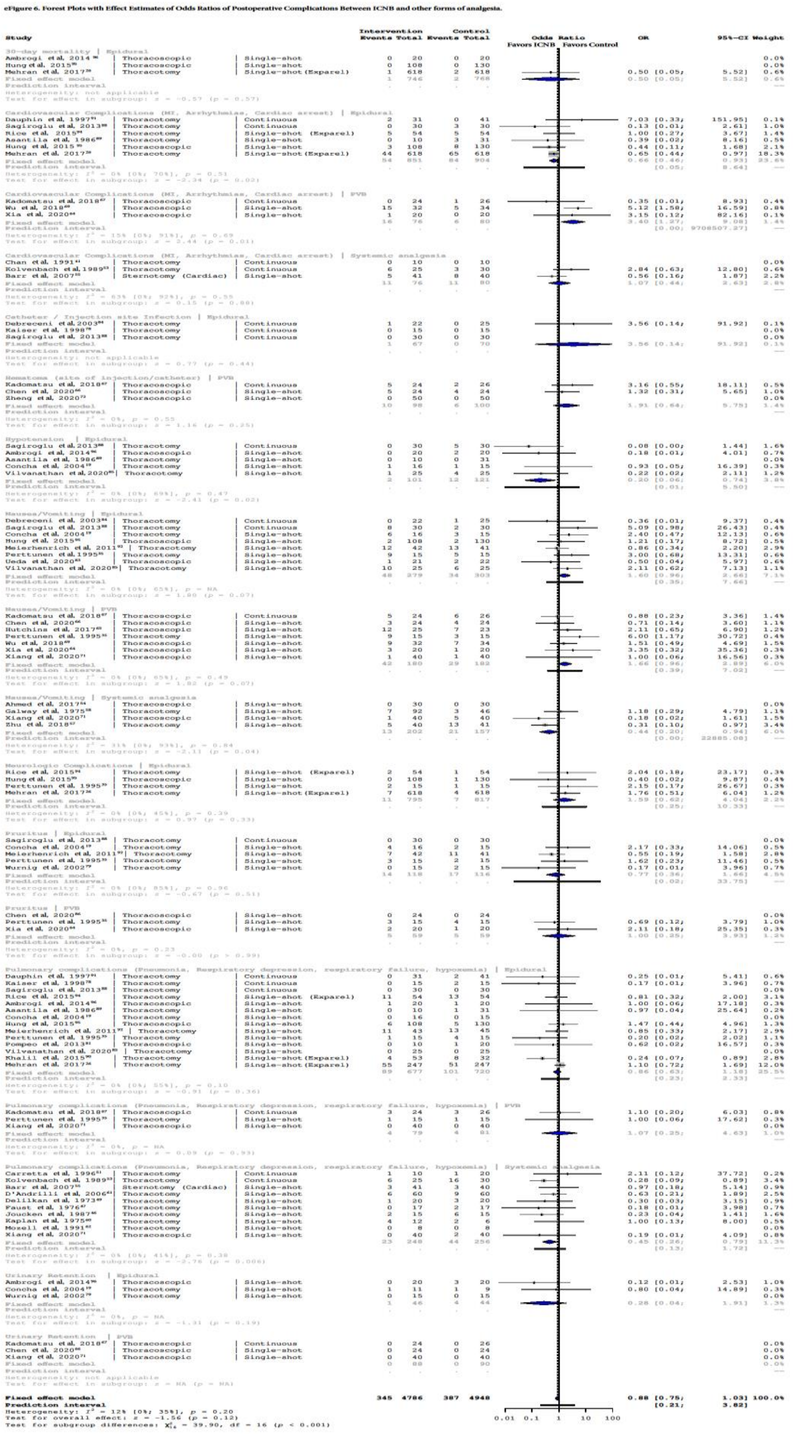

© 2021 Guerra-Londono CE et al. *JAMA Network Open*.

eFigure 7. Forest Plots With Effect Estimates of Odds Ratios for Postoperative Complications Between ICNB and Other Forms of Analgesia: Subgroup Analysis by Type of Surgery (double-click to open PDF)

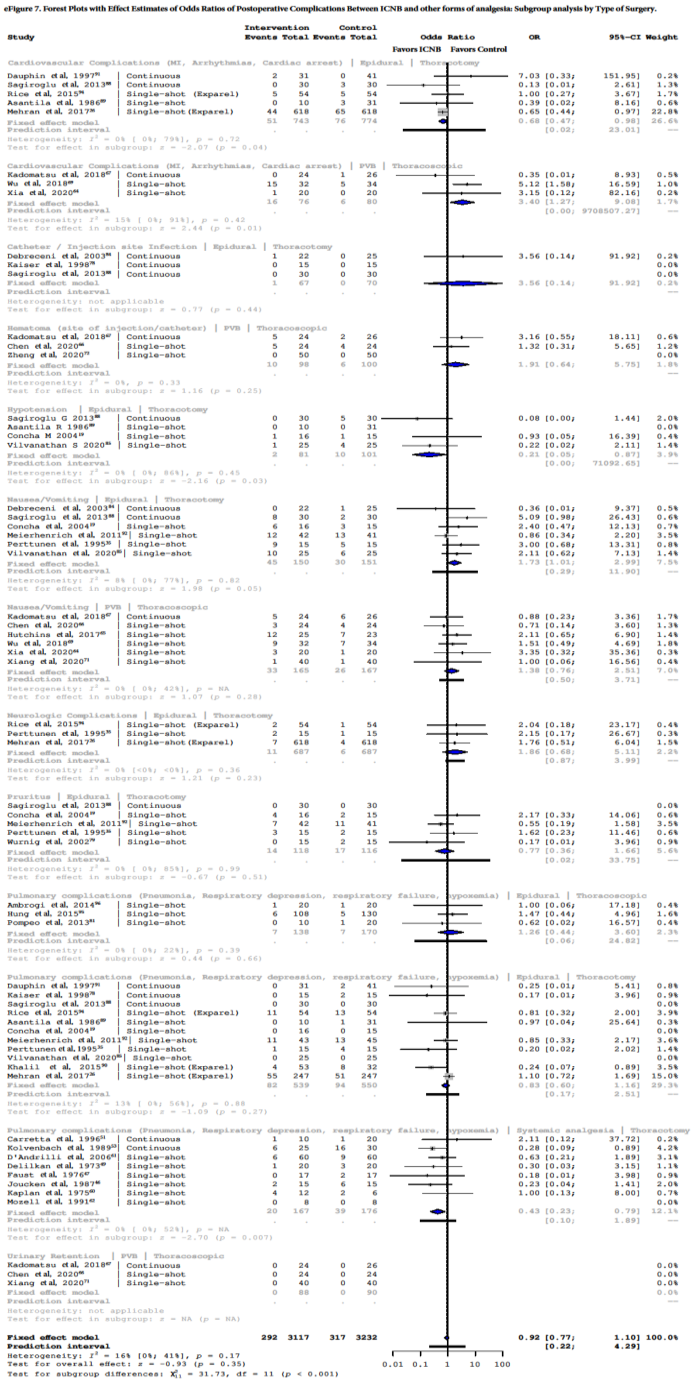

© 2021 Guerra-Londono CE et al. *JAMA Network Open*.

eFigure 8. Forest Plots With Effect Estimates of Mean Differences in Postoperative Pulmonary Function Between ICNB and Other Forms of Analgesia (double-click to open PDF)

eFigure 8. Forest Plots with Effect Estimates of Mean Differences of Postoperative Pulmonary Function Between ICNB and other forms of analgesia.

Study			Intervention Total	Intervention Mean	Intervention SD	Control Total	Control Mean	Control SD	MD	95%-CI	Weight
**FEV1 25–48 hrs | Systemic analgesia**											
de la Rocha et al, 1984[33]	Thoracotomy	Single-shot	5	37.80	19.60	10	42.60	10.23	−4.80	[−23.11; 13.51]	0.4%
Mozell et al, 1991[62]	Thoracotomy	Single-shot	8	88.90	22.40	8	39.00	12.20	49.90	[32.22; 67.58]	0.5%
Toledo-Pereyra et al, 1979[42]	Thoracotomy	Single-shot	10	71.21	3.68	10	56.06	4.33	15.15	[11.63; 18.67]	11.6%
Fixed effect model			23			28			15.75	[12.35; 19.14]	12.5%
Prediction interval			.			.				[−297.61; 337.36]	--
Heterogeneity: $I^2 = 90\%$ [72%; >96%], $p < 0.001$											
Test for effect in subgroup: $z = 9.09$ ($p < 0.001$)											
**FEV1 49–72 hrs | Systemic analgesia**											
Ghafouri et al, 2008[52]	Thoracotomy	Repeated intercostal block	25	77.00	43.00	25	57.00	53.00	20.00	[−6.75; 46.75]	0.2%
de la Rocha et al, 1984[33]	Thoracotomy	Single-shot	5	45.40	19.30	10	47.05	10.47	−1.65	[−19.77; 16.47]	0.4%
Mozell et al, 1991[62]	Thoracotomy	Single-shot	8	101.80	21.22	8	51.70	8.91	50.10	[34.15; 66.05]	0.6%
Toledo-Pereyra et al, 1979[42]	Thoracotomy	Single-shot	10	72.72	3.03	10	53.67	3.89	19.05	[15.99; 22.11]	15.4%
Fixed effect model			48			53			19.57	[16.63; 22.52]	16.6%
Prediction interval			.			.				[−62.01; 106.32]	--
Heterogeneity: $I^2 = 85\%$ [61%; >94%], $p = 0.44$											
Test for effect in subgroup: $z = 13.03$ ($p < 0.001$)											
**FEV1 7–24 hrs | Systemic analgesia**											
de la Rocha et al, 1984[33]	Thoracotomy	Single-shot	5	36.20	22.30	10	35.35	14.57	0.85	[−20.68; 22.38]	0.3%
Mozell et al, 1991[62]	Thoracotomy	Single-shot	8	76.30	7.92	8	32.20	16.13	44.10	[31.65; 56.55]	0.9%
Toledo-Pereyra et al, 1979[42]	Thoracotomy	Single-shot	10	80.30	4.55	10	61.90	4.55	18.40	[14.41; 22.39]	9.1%
Fixed effect model			23			28			20.19	[16.45; 23.93]	10.3%
Prediction interval			.			.				[−236.53; 281.38]	--
Heterogeneity: $I^2 = 89\%$ [70%; >96%], $p < 0.001$											
Test for effect in subgroup: $z = 10.58$ ($p < 0.001$)											
**FEV1 72 hrs | Systemic analgesia**											
de la Rocha et al, 1984[33]	Thoracotomy	Single-shot	5	46.80	18.60	10	47.70	9.91	−0.90	[−18.32; 16.52]	0.5%
Mozell et al, 1991[62]	Thoracotomy	Single-shot	8	117.90	45.56	8	56.40	15.84	61.50	[28.08; 94.92]	0.1%
Toledo-Pereyra et al, 1979[42]	Thoracotomy	Single-shot	10	80.95	3.90	10	60.82	4.12	20.13	[16.61; 23.65]	11.6%
Fixed effect model			23			28			19.75	[16.32; 23.18]	12.3%
Prediction interval			.			.				[−281.58; 325.75]	--
Heterogeneity: $I^2 = 83\%$ [46%; >94%], $p < 0.001$											
Test for effect in subgroup: $z = 11.29$ ($p < 0.001$)											
**FVC 25–48 hrs | Systemic analgesia**											
Carretta et al, 1996[51]	Thoracotomy	Continuous	10	45.89	8.97	20	46.55	11.81	−0.66	[−8.26; 6.94]	2.5%
Mozell et al, 1991[62]	Thoracotomy	Single-shot	8	87.20	23.20	8	39.70	8.49	47.50	[30.38; 64.62]	0.5%
Toledo-Pereyra et al, 1979[42]	Thoracotomy	Single-shot	10	67.39	3.05	10	58.26	3.05	9.13	[6.46; 11.80]	20.2%
Fixed effect model			28			38			8.89	[6.39; 11.38]	23.1%
Prediction interval			.			.				[−224.04; 255.69]	--
Heterogeneity: $I^2 = 92\%$ [80%; >97%], $p < 0.001$											
Test for effect in subgroup: $z = 6.98$ ($p < 0.001$)											
**FVC 7–24 hrs | Epidural**											
Debreceni et al, 2003[84]	Thoracotomy	Continuous	22	41.00	20.00	25	42.00	13.00	−1.00	[−10.79; 8.79]	1.5%
Kaiser et al, 1998[78]	Thoracotomy	Continuous	15	49.73	23.22	15	40.74	12.15	8.99	[−4.27; 22.25]	0.8%
Ueda et al, 2020[83]	Thoracoscopic	Single-shot	21	60.30	17.10	22	54.90	12.30	5.40	[−3.54; 14.34]	1.8%
Fixed effect model			58			62			3.78	[−2.13; 9.69]	4.1%
Prediction interval			.			.				[−30.86; 38.42]	--
Heterogeneity: $I^2 = 0\%$ [0%; >87%], $p < 0.001$											
Test for effect in subgroup: $z = 1.25$ ($p = 0.21$)											
**FVC 7–24 hrs | Systemic analgesia**											
Carretta et al, 1996[51]	Thoracotomy	Continuous	10	38.07	14.13	20	41.98	11.77	−3.91	[−14.07; 6.25]	1.4%
Mozell et al, 1991[62]	Thoracotomy	Single-shot	8	75.80	10.32	8	36.70	16.50	39.10	[25.61; 52.59]	0.8%
Toledo-Pereyra et al, 1979[42]	Thoracotomy	Single-shot	10	76.08	3.26	10	65.21	3.04	10.87	[8.11; 13.63]	18.9%
Fixed effect model			28			38			10.95	[8.34; 13.57]	21.1%
Prediction interval			.			.				[−227.83; 256.97]	--
Heterogeneity: $I^2 = 92\%$ [80%; >97%], $p = 0.003$											
Test for effect in subgroup: $z = 8.21$ ($p < 0.001$)											
**Fixed effect model**			**231**			**275**			**14.24**	**[13.04; 15.44]**	**100.0%**
**Prediction interval**										**[−3.29; 35.76]**	
Heterogeneity: $I^2 = 89\%$ [85%; 92%], $p < 0.001$											
Test for overall effect: $z = 23.26$ ($p < 0.001$)											
Test for subgroup differences: $\chi^2_6 = 68.79$, df = 6 ($p < 0.001$)											

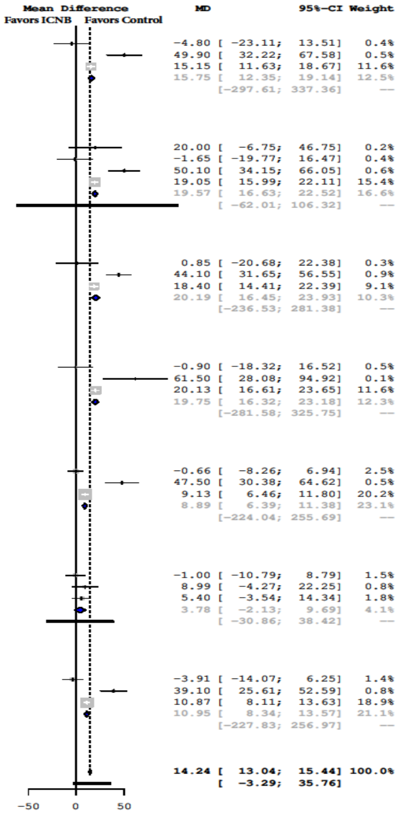

© 2021 Guerra-Londono CE et al. *JAMA Network Open*.

eFigure 9. Forest Plots With Effect Estimates of Mean Differences in Postoperative Pulmonary Function Between ICNB and Other Forms of Analgesia: Subgroup Analysis by Type of Surgery (double-click to open PDF)

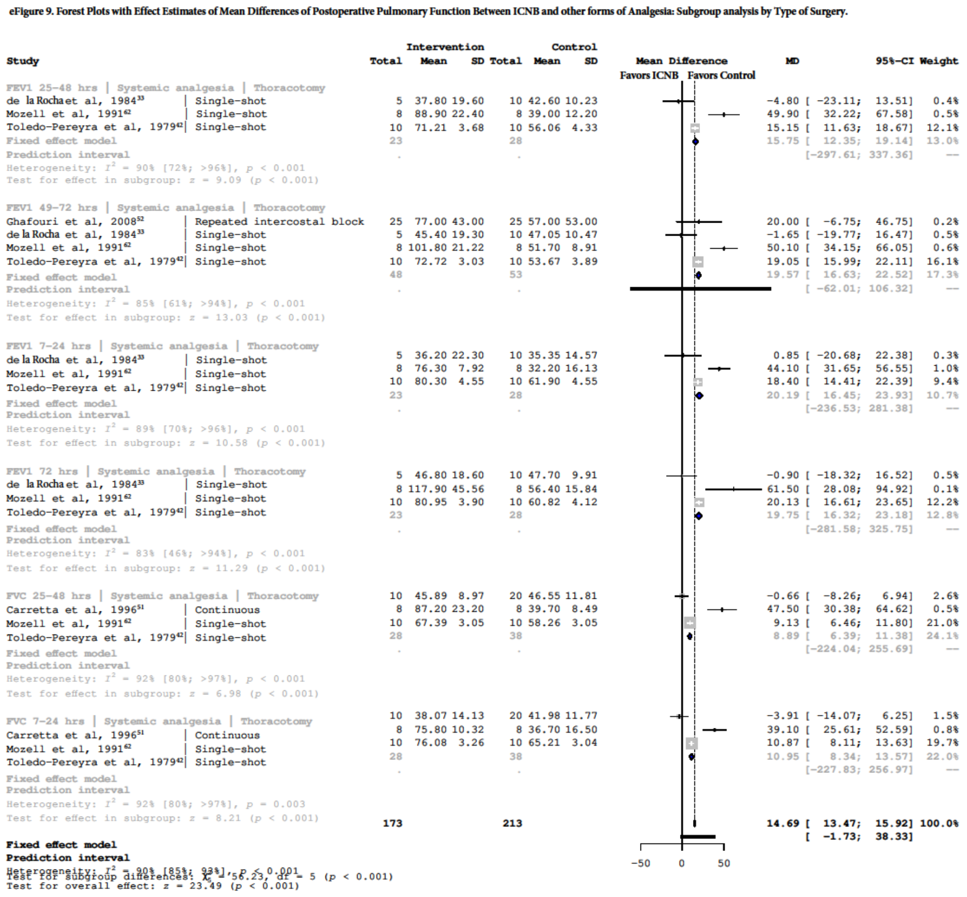

© 2021 Guerra-Londono CE et al. *JAMA Network Open*.

eFigure 10. Forest Plots With Effect Estimates of Mean Differences in Length of Stay (Hours) Between ICNB and Other Forms of Analgesia (double-click to open PDF)

eFigure 10. Forest Plots with Effect Estimates of Mean Differences of Length of Stay (hours) Between ICNB and other forms of analgesia.

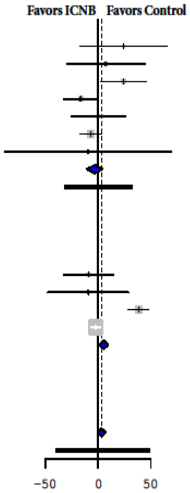

Study			Intervention Total	Intervention Mean	Intervention SD	Control Total	Control Mean	Control SD	MD	95%-CI	Weight
Hospital LOS (hrs) | Epidural											
Debreceni et al, 2003[84]	Thoracotomy	Continuous	22	192.00	72.00	25	168.00	72.00	24.00	[−17.25; 65.25]	0.8%
Kaiser et al, 1998[78]	Thoracotomy	Continuous	15	372.00	52.80	15	364.80	50.40	7.20	[−29.74; 44.14]	1.0%
Sagiroglu et al, 2013[88]	Thoracotomy	Continuous	30	312.00	48.00	30	288.00	36.00	24.00	[2.53; 45.47]	2.8%
Hung et al, 2015[95]	Thoracoscopic	Single-shot	108	127.20	55.20	130	144.00	72.00	−16.80	[−32.97; −0.63]	5.0%
Perttunen et al, 1995[35]	Thoracotomy	Single-shot	15	175.20	36.00	15	175.20	36.00	0.00	[−25.76; 25.76]	2.0%
Pompeo et al, 2013[81]	Thoracoscopic	Single-shot	10	28.80	9.60	20	36.00	19.20	−7.20	[−17.51; 3.11]	12.3%
Ueda et al, 2020[83]	Thoracoscopic	Single-shot	21	259.20	139.20	22	268.80	122.40	−9.60	[−88.09; 68.89]	0.2%
Fixed effect model			221			257			−3.38	[−10.75; 4.00]	24.1%
Prediction interval			.			.				[−31.34; 32.40]	--
Heterogeneity: I^2 = 48% [0%; >78%], p = 0.07											
Test for effect in subgroup: z = −0.90 (p = 0.37)											
Hospital LOS (hrs) | PVB											
Hutchins et al, 2017[65]	Thoracoscopic	Single-shot	25	29.28	39.36	23	38.40	43.44	−9.12	[−32.64; 14.40]	2.4%
Perttunen et al, 1995[35]	Thoracotomy	Single-shot	15	175.20	36.00	15	184.80	66.00	−9.60	[−47.65; 28.45]	0.9%
Xia et al, 2020[64]	Thoracoscopic	Single-shot	20	96.00	9.60	20	57.60	19.20	38.40	[28.99; 47.81]	14.8%
Xiang et al, 2020[71]	Thoracoscopic	Single-shot	40	76.80	12.00	40	79.20	9.60	−2.40	[−7.16; 2.36]	57.8%
Fixed effect model			100			98			5.27	[1.11; 9.42]	75.9%
Prediction interval			.			.				[−116.15; 128.49]	--
Heterogeneity: I^2 = 95% [90%; >97%], p < 0.001											
Test for effect in subgroup: z = 2.48 (p = 0.01)											
**Fixed effect model**			**321**			**355**			**3.18**	**[−0.44; 6.80]**	**100.0%**
**Prediction interval**										**[−40.22; 48.90]**	
Heterogeneity: I^2 = 87% [78%; 92%], p < 0.001											
Test for overall effect: z = 1.72 (p = 0.08)											
Test for subgroup differences: χ^2_1 = 4.01, df = 1 (p = 0.05)											

© 2021 Guerra-Londono CE et al. *JAMA Network Open*.

eFigure 11. Forest Plots With Effect Estimates of Mean Differences in Length of Stay (Hours) Between ICNB and Other Forms of Analgesia: Subgroup Analysis by Type of Surgery (double-click to open PDF)

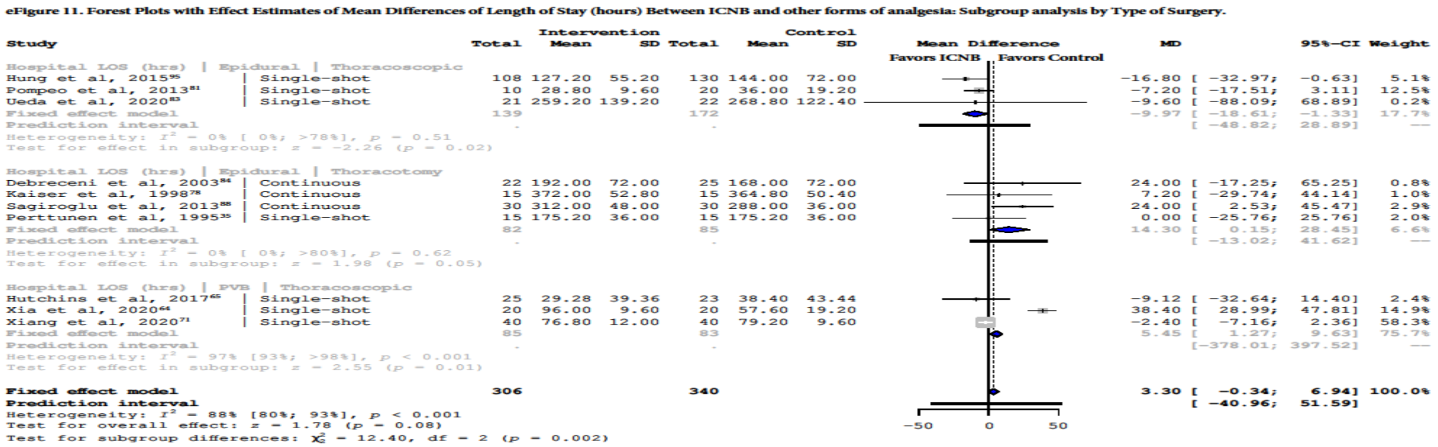

© 2021 Guerra-Londono CE et al. *JAMA Network Open*.
